# The Role of Carbon Fibers in FFF 3D Printed Composites: A Comprehensive Review on the Effect on Mechanical Properties, Fracture Toughness, and Tribological Performance

**DOI:** 10.3390/polym18192369

**Published:** 2026-09-28

**Authors:** Lorenzo De Noni, Hengxi Chen

**Affiliations:** 1 Polymer Technology Consortia, Department of Materials Science and Engineering, Texas A&M University, College Station, TX 77843, USA; 2 Department of Plastics Engineering, University of Massachusetts Lowell, Lowell, MA 01854, USA

**Keywords:** Fused Filament Fabrication, carbon fibers, carbon fiber-reinforced composites, mechanical properties, tribological properties, fracture behavior

## Abstract

Polymeric composites reinforced with short or continuous carbon fibers offer exceptional mechanical and tribological properties that outperform their unfilled counterparts. The combination of these excellent properties with the possibility to manufacture custom-made composites with complex shapes and geometry is unique; therefore, additive manufacturing has recently emerged as an intriguing manufacturing technique combining these two features. Specifically, Fused Filament Fabrication (FFF) has attracted significant academic and industrial attention owing to its operational simplicity, cost-effectiveness, and compatibility with a wide range of thermoplastic matrices. This review provides a comprehensive overview of FFF-printed carbon-fiber reinforced composites, focusing on strategies to optimize both their mechanical and tribological properties. The first part introduces the core aspects of FFF technology and highlights how the printing process is altered by the incorporation of carbon fibers. The central part systematically points out the benefits of utilizing short or continuous carbon fibers on the mechanical and tribological performance of the composites. The final section addresses advanced post-processing techniques designed to mitigate typical printing issues, followed by an analysis of current research trajectories. Overall, this review examines the current literature to provide a state-of-the-art summary, highlighting the manufacturing possibilities, inherent limitations, and new research frontiers of FFF-printed carbon fiber-reinforced polymer composites.

## 1. Introduction

The idea of creating physical objects from geometrical representation, depositing the material layer-by-layer, was first introduced commercially by Hull [1] in the 1980s and has since attracted significant interest and development due to its ability to manufacture complex geometries. It is nowadays known as “additive manufacturing” or “3D printing”. During the last three decades, several AM technologies have been developed, the most common and well-developed being material extrusion processes such as Fused Filament Fabrication (FFF) (also known as Fused Deposition Modeling, FDM); additional widely used polymer additive manufacturing processes include inkjet printing [2,3], vat polymerization [4], powder bed fusion [5], selective laser sintering [6]. The use of FFF for polymer 3D printing is widespread, and its popularity stems from its low entry cost, simple user operation, the variety of available filaments, decent part quality and repeatability, and the relative simplicity of the process compared to powder or resin-based systems. The technology is seeing rapid adoption within academic circles, industrial sectors, and the hobbyist community. Another significant advantage of FFF is the wide array of available materials [7,8], such as acrylonitrile-butadiene styrene (ABS) [9], polylactide (PLA) [10,11,12,13], polyamide [14], polycarbonate [15], Polyethylene Terephthalate Glycol (PETG) [16], and engineering-grade polymers such as polyether ether ketone (PEEK) [17], polyetherketoneketone (PEKK) [18]. To reduce the impact of plastic pollution, bio-based polymers, such as polyhydroxyalkanoates [19], are also being studied for 3D printing applications. Beyond conventional polymers, FFF can be used for multi-material and fiber-reinforced composite printing [12,20,21,22,23]. Carbon-fiber-reinforced, either short or long, composites have gained increasing popularity, and the advent of 3D printing enables the fabrication of complex geometries that are difficult to achieve using conventional manufacturing methods. Three main approaches have been reported for incorporating fibers into FFF-printed composites [24], as illustrated schematically in Figure 1:

- The fiber is incorporated before the printing process; in other words, the filament itself is a composite;
- The fiber is incorporated within the print head, meaning the two materials are combined as they pass through the extruder;
- The fiber is incorporated directly into the component, thus requiring two or more independent extruders, each equipped with its own nozzle.

In this context, the incorporation of carbon fibers in printed materials offers several advantages, including tailored and sometimes enhanced mechanical (such as tensile and flexural strength, compression resistance, and impact resistance) [25,26], tribological [27], thermal [28], and electrical performance [29], while simultaneously enabling the production of intricate shapes that would otherwise be nearly impossible to manufacture. Several challenges still must be addressed, such as developing a proper matrix, fibers, and fiber treatment to allow 3D printing of such composite components. One of the most common reasons for macroscopic failures in 3D printed carbon fiber reinforced composites is insufficient carbon fiber impregnation and poor interface adhesion between the fibers and the matrix [30]. The development of voids between fibers and matrix adds additional challenges for property enhancement in carbon fiber reinforced composites. Research is currently focusing on:

1. Process Optimization: Enhancing printing equipment and part quality by mitigating common defects, such as void formation, warping, and poor fiber-matrix interfacial adhesion.
2. Performance Enhancement: Optimizing the mechanical, tribological, thermal, and electrical properties of components through advanced material selection and process control, with the ultimate goal of achieving parity with conventional manufacturing standards (e.g., injection molding).
3. Sustainability and Circularity: Investigating the recyclability of 3D-printed parts and the integration of recycled feedstocks to minimize waste and repurpose end-of-life components.

This review investigates the role of carbon fibers on 3D printed composites and their effect on the mechanical and tribological performance of the printed component. The number of research studies is growing rapidly, but the data and information are still scattered, therefore the present literature review aims at collecting and organizing the data in a manner that highlights the beneficial effect of the carbon fibers in 3D printing processes. It is worth noting that mechanical and tribological properties are a function of the materials used (matrix, fibers, types of fibers, fiber treatment, etc.) but also of printing parameters such as nozzle size, layer thickness, infill density and pattern, building orientation, raster angle, printing speed, printing temperature, bed temperature, etc. A major part of these parameters is material-independent or has the same effect regardless of the material used; therefore, they cannot be strictly related to the use of carbon fibers in 3D printed composites. Therefore, the present review will try to separate the contribution of the printing procedure from the beneficial effect of carbon fibers on the mechanical and tribological properties of 3D printed carbon fiber reinforced composites. The review first addresses the generic effect of FFF process parameters and fiber-specific printing challenges, then examines the effect of carbon fibers on tensile, fracture, and tribological performance, and closes with post-processing strategies and future research directions.

## 2. Generic Effect of 3D Printing Process Parameters and 3D Printing Manufacturing Issues

### 2.1. Key Processing Parameters

The following parameters affect FFF-printed composites regardless of fiber content; understanding their baseline effects is necessary to isolate the specific contribution of carbon fibers in the following sections.

#### 2.1.1. Effect of Infill Density

Printing parameters such as infill density and infill pattern affect the performance of FFF-printed CFRPs. Infill pattern and its density, shown in Figure 2 [31], largely impact the properties of the printed component [32]: low infill density leads to larger voids and reduces the cross-sectional resistance to mechanical stresses, thus worsening the mechanical response of the printed components [33,34]. Vice versa, higher infill density generally leads to higher stiffness and strength but involves slower printing. The same principle can be applied to fiber reinforced composites, the effect being less important thanks to the reinforcing effect of carbon fibers [35,36].

#### 2.1.2. Effect of Layer Thickness

Layer thickness also has a crucial effect: thicker layers reduce printing time but usually lead to increased porosity among layers and may not lead to full material melting during the extrusion process. Also, the surface finishing is often rougher. On the other hand, thinner layers improve surface finish and interlayer bonding due to a higher contact area. All of these causes can impact mechanical strength due to a larger number of defects and non-uniform polymer properties [38].

#### 2.1.3. Effect of Build Orientation/Raster Angle

3D printing allows specimens to be built in on-edge, flat, and upright orientations (Figure 3a, Figure 3b and Figure 3c, respectively), and the chosen orientation strongly affects mechanical performance due to the anisotropy created by layer-by-layer deposition [39,40]. This effect is even more pronounced in carbon fiber-reinforced 3D printed composites. During composite printing, the alignment of short and continuous fibers can be guided by the shear stress generated at the extrusion nozzle. This effect promotes the orientation of carbon fibers along the printing direction, potentially improving mechanical properties such as tensile strength [41,42]. Given that the fibers are highly aligned along the individual beads, the angle at which these beads are deposited determines the anisotropy of the part. This parameter, known as the raster angle or raster orientation, defines the angle between the *x*-axis of the build table and the strand deposition along the raster tool path [43], generally ranging from 0^°^ to 90^°^. In terms of internal defects, FFF-printed CFRPs suffer heavily from inter-bead porosity that reduces mechanical properties. When the raster angle varies, the orientations of both the short fibers and the inter-bead pores shift simultaneously, which introduces anisotropy into the mechanical performance. Iyer et al. [44] observed a distinct decreasing trend in tensile properties with an increasing raster angle in FFF-printed, SCF-reinforced acrylonitrile butadiene styrene (ABS) composites. As shown in Figure 4a,b, the tensile strength decreased by 22% (from 31.5 MPa to 24.6 MPa) and the modulus decreased by 35% (from 4.6 GPa to 3.0 GPa) when the raster angle shifted from 0° to 15°. As the raster angle further increased from 0^o^ to 90^o^, an increase in inter-bead porosity was observed (Figure 4c), which may have contributed to the reduction in the tensile properties of the specimens.

#### 2.1.4. Effect of Bed Temperature

Bed temperature controls the cooling rate of the printed component, reducing warping and residual stress while promoting interlayer adhesion. For amorphous polymers, higher bed temperatures approaching T_g_ enhance chain mobility and interdiffusion across bead interfaces, improving interlayer bonding. For semi-crystalline polymers such as PA and PEEK, the relevant parameter is the crystallization temperature T_c_: a higher bed temperature slows the cooling rate, allowing the melt to spend more time in the crystallization window between T_m_ and T_g_, which promotes higher crystallinity and more ordered microstructure. In both cases, controlled thermal management during deposition improves mechanical and tribological performance.

#### 2.1.5. Effect of Printing Temperature

Printing temperature is a crucial parameter: higher printing temperatures generally improve the interlayer adhesion and polymer diffusion between deposited filaments; however, too high temperatures can degrade the polymer, leading to weaker mechanical strength. For carbon reinforced polymers, temperature is crucial as it influences the impregnation quality of the reinforcing fibers. Higher temperatures usually lead to improved adhesion between polymer and fibers. For instance, Tian et al. [38] showed that for CF/PLA composites, increasing printing temperature from 180 to 240 °C progressively improved modulus and flexural strength through better fiber impregnation, while temperatures above 220 °C degraded surface quality due to polymer overflow, illustrating the trade-off inherent to printing temperature optimization.

#### 2.1.6. Effect of Printing Speed

Printing speed plays a significant role: faster printing reduces bonding time between filaments, thus leading to weaker interlayer adhesion. Slower printing allows better interlayer adhesion but may cause overheating or warping and increase production time. This phenomenon is also crucial in fiber-reinforced composites. For instance, it was shown [38] that for PLA/CF composites, printed with a velocity between 60 and 160 mm/min, increased printing velocity resulted in a drop in fiber content while increasing the internal pressure of the liquefier and the contact pressure between the nozzle and the deposited layer. Due to the contradictory effects of these observations on the composite’s mechanical properties, the relationship between filament feed rate and mechanical properties is unclear. A feed rate of 80–100 mm/min was proposed to balance these opposing effects, as also observed elsewhere [45].

Overall, the representative quantitative effects of the key FFF processing parameters discussed above on mechanical properties are summarized in Table 1. The quantitative results in Table 1 indicate that the mechanical performance of FFF-printed CFRPs is particularly sensitive to raster angle, printing temperature, and printing speed. Raster angle directly determines the alignment of the reinforcing fibers relative to the loading direction and therefore strongly affects load-transfer efficiency and mechanical anisotropy. Printing temperature and printing speed primarily influence melt flow, fiber impregnation, interfacial wetting, and interlayer consolidation, thereby controlling the quality of stress transfer within the printed structure. Infill density and layer thickness also produce substantial changes in the resulting mechanical properties, but their effects are largely associated with changes in the effective load-bearing area, bead geometry, porosity, and the number of interlayer interfaces. In comparison, bed temperature shows a relatively minor influence within the investigated range and may therefore be regarded as a secondary processing factor. However, the relative importance of these processing parameters depends on the material system, material structure and architecture, and the property being evaluated.

## 3. Process Challenges in FFF Printing in Carbon Fiber-Reinforced Composites

While the process parameters described above govern the general quality of any FFF-printed part, the incorporation of carbon fibers introduces additional manufacturing challenges that are specific to fiber-reinforced systems and that complement the process-property relationships discussed above.

### 3.1. Nozzle Clogging

One of the major current challenges in 3D printing of carbon fiber-reinforced composite is to increase the fiber content, beneficial for the properties of the component, while minimizing unwanted effects, such as nozzle clogging. Both fiber content and fiber shape/length matter for the clogging phenomenon. Increasing fiber content enhances contact between fibers and prevents their reorientation, thereby increasing the likelihood that fiber agglomerates produce clogs [50]. The mechanism behind such phenomena is mainly driven by the presence of strongly misoriented fibers within the nozzle, as shown in Figure 5 [51]. While most fibers were strongly aligned with the extrusion direction, misoriented fibers resisted the strong alignment kinetics associated with the nozzle flow conditions, thus leading to nozzle clogging. Zhang et al. [51] demonstrated both experimentally and numerically (through computational fluid dynamics simulation) that low volume fraction and short fibers are beneficial for the polymer-fibers flow within the nozzle, thus reducing the chances of nozzle clogging. In this sense, print head type [52], volume fraction and fiber length [51] appears to be the key factors leading to nozzle clogging. To the best of our knowledge, no research has focused on the effect of the fiber shape or length ratio.

### 3.2. Nozzle Abrasive Wear

As mentioned previously, the primary mechanical degradation in extruding CFRP filaments originates from the difference in hardness between carbon fibers and the metallic nozzle. Standard brass nozzles, although favored for their high thermal conductivity, possess a Mohs hardness significantly lower than that of CFs. These microscale CFs act as abrasive particles, continuously scouring the internal walls and the nozzle during the high-pressure extrusion process. As shown in Figure 6a,b [53], the mechanical wear further tends to progressively degrade the nozzle geometry over time. Moreover, surface quality progressively deteriorates, evidenced by macroscopic voids, defects, and cracks, with increasing cumulative printing time (Figure 6c,d).

This abrasive interaction leads to two distinct morphological changes: (1) internal bore expansion, and (2) tip flattening. As the nozzle diameter increases, the flow resistance decreases, therefore resulting in unintended over-extrusion and a loss of dimensional precision in the extrudates. Furthermore, the abrasion of the nozzle tip roughens the surface finish quality of the printed part and compromises the compaction pressure required for interlayer bonding [53]. To mitigate this mechanical degradation, researchers have transitioned toward hardened materials, such as hardened steel, tungsten carbide, or ruby-tipped nozzles. However, these alternatives often introduce a trade-off in thermal management, as their low thermal conductivity can necessitate higher processing temperatures to maintain consistent melt flow.

### 3.3. Die Swell and Fiber Orientation Within the Melt

As the composite melt exits the nozzle orifice, it transitions from a highly constrained, high-pressure environment to a free-surface state. This transition triggers two simultaneous and competing phenomena: extrudate swell (die swell) and the final orientation state of the fibers, both of which govern the dimensional accuracy and mechanical performance of the printed part.

Die swell in SCF-reinforced polymers is primarily driven by the entropic relaxation of polymer chains that were previously stretched and oriented during the convergent flow within the nozzle. When the geometric constraint of the nozzle wall is removed, the stored elastic energy is rapidly released, causing the extrudate to expand transversally. Interestingly, the addition of rigid CFs typically attenuates the magnitude of this die swell compared to neat polymers. This suppression occurs because rigid fibers cannot store elastic energy. Instead, they act as physical constraints that hinder the transverse relaxation of the surrounding polymer matrix. Nonetheless, excessive die swell can still induce “over-extrusion” artifacts, where the deposited bead dimensions exceed the programmed toolpath, thereby compromising the geometric tolerance of the printed component.

Given that extrudate swell represents the last source of severe fiber orientation change within the polymer melt during extrusion, it exerts a significant influence on the final fiber orientation state and, consequently, the mechanical properties of the deposited FFF bead [54]. The shear-induced alignment of SCFs during extrusion represents a significant advantage of FFF, as it enables the realization of “tailored anisotropy”. As the melt accelerates through the contraction zone of the nozzle, the coupled effects of high shear rates and extensional flow force the fibers to align parallel to the extrusion direction. The efficiency of this alignment process is highly dependent on both the fiber aspect ratio and the nozzle contraction ratio. Research shows that a higher degree of fiber alignment results in superior tensile strength along the printing path, but it introduces significant vulnerability in the transverse direction. Additionally, upon exiting the orifice, this ideal alignment can be slightly perturbed, particularly at the outer boundaries of the extruded bead, due to the sudden change in velocity profile and the resulting “fountain flow” effect. The complex interplay between die swell and fiber orientation determines the bead aspect ratio (width-to-height). For continuous carbon fiber systems, die swell along the continuous fiber axis is relatively negligible due to the permanent axial constraint. However, elastic swelling can still manifest within the surrounding thermoplastic cladding, which directly influences the inter-bead consolidation and bonding quality between adjacent paths.

### 3.4. Fiber Distribution and Breakage

Zhang et al. [55] showed that when fiber reinforced filaments are printed with turning angle or curvature, a situation commonly encountered in printing shapes, numerous printing defects are observed, including path error, fiber twisting, folding and misalignment, caused by the excessive tensile force from the nozzle, which may dramatically lead to fiber breakage (Figure 7) and significant change in the layer thickness.

### 3.5. Micro-Voids Formation Within Fiber and Matrix Filament

Micro-void formation, both between printed layers and at the fiber-matrix interface, is one of the most critical defects in FFF-produced composites. While interlayer voids can be partially controlled by adjusting process parameters (e.g., layer thickness), voids between fibers and matrix are often unavoidable. Ning et al. [57] demonstrated that the presence of carbon fibers may result in different pore types, specifically gas-generated pores, that are mainly generated during the fabrication of the feedstock filament by extrusion. When depositing the materials using FFF, various carbon fiber distributions in the filament resulted in inconsistent fusion of the layers, as shown in Figure 8 [58]. Varying the carbon fiber content results in either a lower or larger amount of porosity, which in turn affects the mechanical performance of the printed component. Similar results were found elsewhere [59,60].

### 3.6. Warpage and Shrinkage

In carbon fiber-reinforced composites, there is a significant mismatch between the thermal conductivity of the fibers and the matrix: after materials deposition, the cooling rates vary across different regions of the printed part, thus resulting in substantial temperature gradients giving rise to thermal stresses and uneven warpage/shrinkage [61], as shown in Figure 9 [62]. Overall, the process challenges in FFF-printed CFRPs can be divided into those that directly affect mechanical performance and those that mainly affect processing stability. Fiber orientation, fiber breakage/distribution, and micro-void formation are the primary factors because they directly govern load transfer, reinforcement continuity, and defect sensitivity. In comparison, nozzle clogging, abrasive wear, and warpage/shrinkage primarily influence process reliability and dimensional accuracy, although they may indirectly deteriorate mechanical performance. These challenges are strongly inter-connected, and therefore optimization requires balancing reinforcement efficiency with processability and defect control.

## 4. Effect of Carbon Fibers on the Mechanical and Tribological Properties of 3D Printed Composites

Although CF reinforcement improves the mechanical properties of FFF-printed composites, performance remains inferior to conventionally manufactured counterparts, primarily due to void formation, poor interlayer adhesion, and the process-dependent fiber orientation state. The following sections address tensile properties, fracture behavior, and tribological performance.

### 4.1. Effect on Tensile Properties

Among various mechanical indices, tensile properties, specifically tensile strength and elastic modulus, are the most used parameters, as they directly reflect the load-bearing capability of the fiber-matrix system. However, this mechanical performance [63] is not merely a function of material formulations but is intrinsically governed by the hierarchical microstructure, namely the geometric differences between short carbon fibers (SCF) and continuous carbon fibers (CCF), the fiber orientation state, and the fiber aspect ratio (L/D).

Compared to neat polymer matrices, CF reinforcement could moderately enhance mechanical properties, though this reinforcement efficiency remains heavily dependent on the content and alignment of the distributed CFs [64]. Importantly, CF reinforcement does not yield isotropic behavior; while the tensile modulus improves across all print orientations, the tensile strength is the highest at 0° orientation, where fibers align with the loading direction. In contrast, strength decreases significantly as the raster angle deviates from the load axis, because interlayer bonds rather than fibers carry an increasing share of the load. This highly anisotropic behavior is driven by the fact that CFs are predominantly aligned along the major extrusion path. For example, Jiang and Smith [64] found that the addition of carbon fiber improves the tensile modulus of PLA, ABS, PETG, across all print orientations. Specifically, the most pronounced improvement in tensile strength was achieved at the 0° print orientation, where the strength reached 50.9 MPa (a 33.2% improvement) for ABS, 68.4 MPa (a 14.0% improvement) for PLA, and 68.3 MPa (a 48.2% improvement) for PETG. Notably, similar reinforcing effects and anisotropic behaviors is also observed for the flexural and compressive properties of FFF-printed CFRPs, where aligning the continuous or short fibers parallel to the principal stress vector yields better mechanical performance [65,66]. Moreover, the adoption of mixed orientations or multi-directional toolpaths for continuous CF represents a promising solution to realize balanced laminates capable of withstanding complex, multiaxial stress states. For instance, implementing a quasi-isotropic stacking sequence (such as [0°/+45°/90°/−45°]) has a more pronounced influence on the flexural properties than on the tensile behavior. This is primarily attributed to improved load sharing between differently oriented layers [67].

Beyond macroscopic fiber alignment, the local reinforcement efficiency is strongly related to the microstructural state of the fibers. For example, mechanical shearing during extrusion significantly damages the fibers and reduces their resulting fiber aspect ratios (L/D). A high fiber aspect ratio (L/D > 10) typically indicates long, thin fibers, which are expected to improve the mechanical properties significantly if good fiber-matrix adhesion is achieved [68]. Furthermore, the overall tensile properties are also dependent on the resulting fiber volume fractions (V_f_) [69,70] and on the quality of fiber wetting during printing [71]. In general, a higher modulus is expected at higher fiber content or high V_f_ [63,72] because SCFs possess a modulus at least an order of magnitude greater than the polymer matrix. However, an increasing fiber content simultaneously increases the proportion of fibers in non-ideal conditions and the number of microstructural defects in the composites [55].

These processing-induced features help explain why the reinforcement efficiency of FFF-printed CFRPs often falls below idealized theoretical predictions. The classical Rule of Mixtures represents an idealized load-sharing condition between the fiber and matrix, whereas modified formulations introduce efficiency factors to account for finite fiber length and non-ideal fiber orientation [73]. Nevertheless, additional FFF-specified defects, including fiber breakage, insufficient interfacial bonding, porosity, and weak interlayer interfaces, can further reduce stress-transfer efficiency and cause experimental properties to deviate from theoretical predictions. In particular, fiber shortening reduces the fiber aspect ratio and effective load-transfer length [68], while poor fiber-matrix wetting or interfacial adhesion can lead to fiber pull-out and interfacial debonding [63,74]. Moreover, inter- and intra-bead voids reduce the effective load-bearing area and act as stress concentrators, whereas insufficient bonding between adjacent rasters and layers introduces weak interfaces that are not accounted for in idealized composite models. The strong orientation dependence inherent to FFF further limits reinforcement efficiency when the fiber direction is not aligned with the applied load. Consequently, while good agreement between theoretical (such as Rule of Mixtures or modified Rule of Mixtures) and experimental measured elastic properties has been reported in previous studies [63,72], tensile strength often deviates substantially from theoretical predictions due to void formation and fiber shortening. Finite Element Analysis (FEA) was also demonstrated to be a useful tool to predict elastic properties of CFRPs [75,76].

### 4.2. Effect on Fracture Behavior

Fracture toughness in FFF-printed CF composites is governed by the interplay between the polymer matrix, the fiber-matrix interface, and the bead-scale architecture, making it sensitive to both material composition and printing parameters.

#### Fracture Characterization: Mode I, Mode II, and Mixed-Mode Fracture Performance

The most important and most frequently misread aspect of fracture in FFF-printed CF composites is that CF addition can either increase or decrease fracture toughness relative to the neat matrix, depending on fiber type, fiber length, and interfacial quality. Mode I (opening) and Mode II (shear) fracture toughness respond differently to CF reinforcement in FFF composites. Under Mode I loading, fiber bridging is the dominant energy-dissipation mechanism, and FFF-printed CCF composites perform comparably to conventionally manufactured counterparts (hot-compression molded) [77,78,79], as shown in Figure 10 [79]. Santos et al. [77,79] and Iragi et al. [80] demonstrated that FFF-printed CCF/polyamide composites achieve Mode I G_IC_ values comparable to hot-press molded counterparts, with fiber bridging and interlayer void growth as the primary crack growth mechanisms. Kong et al. [78] confirmed these findings for PA composites, and Ogaili et al. [81] showed that in SCF/PLA, fiber bridging, fiber pull-out and crack deflection jointly improve fracture resistance relative to neat PLA, indicating that even short fibers can toughen the matrix. In fact, the irregular interlayer surface topology, a consequence of bead geometry and void formation, forces the crack to navigate a tortuous path, which contributes additional energy dissipation beyond simple bridging. Conversely, Young et al. [82] observed the opposite behavior in SCF/ABS: the addition of fibers can sometimes lead to increased porosity at the interface and poor wetting between fibers and matrix, potentially acting as “brittle inclusions”, rather than “bridging” elements, which leads to a reduction of fracture toughness with respect to the neat system, as also shown elsewhere [83]. The practical implication is clear: CF addition to FFF composites carries a genuine risk of embrittlement if interfacial quality and fiber length are not controlled. Surface treatment is therefore not merely beneficial but necessary: the quality of the fiber-matrix bond can be deemed as the critical bottleneck [45]. Liu et al. [45] demonstrated this directly: applying a compatible polyimide sizing to CCF in a PA6 matrix increased ILSS by 42.2% and shifted the failure mechanism from large-scale pull-out to fiber cut-off.

Under Mode II loading, the same irregular interlayer surface can become a critical factor. Iragi et al. [80] showed that the FFF interlayer morphology does not activate efficient matrix shearing, and G_IIC_ is consequently lower than conventional laminates. Kong et al. [78] confirmed that under mixed-mode conditions, crack propagation tortuosity enhances energy absorption, but propagation stability is reduced. The asymmetry between Mode I and Mode II performance is a structural consequence of the bead-layer architecture and cannot be eliminated by material optimization alone, thus it requires geometric or process-level design strategies (e.g., varying raster angle, reinforcing perimeter walls).

Other printing parameters modulate these effects directly. Somireddy et al. [85] showed that thinner layers improve tensile strength but do not consistently improve interlaminar fracture properties, because the associated changes in thermal history at the interface can reduce local crystallinity or introduce residual stresses that offset the benefit of reduced void content. Papon and Haque [86,87] demonstrated that square-profile nozzles reduce void content and improve bead spreading, improving fracture resistance by addressing the geometric root cause of interlayer weakness. Gao et al. [88] showed that maximizing infill density and the number of reinforced perimeter walls is essential for fully utilizing fiber strength before premature matrix failure. FEA has been used to validate experimentally observed failure modes and to map fiber orientation effects on stress distribution at the crack tip [81,89,90], providing a computational framework for structural design of FFF-printed components. These works showed the importance of tuning printing parameters although none have been systematically investigating their effect on the fracture toughness of carbon fiber reinforced composites. This remains an open area for future research.

### 4.3. Effect on the Tribological Properties

Tribological performance, encompassing friction, wear, hardness, and scratch resistance [91,92,93,94,95,96,97,98], is governed by two contributions. The adhesive component depends on surface energy and junction strength between contacting surfaces. Higher surface energy increases junction strength between surfaces, promoting transfer film formation, debris generation, and adhesive wear. Lower surface energy reduces adhesion and shifts the dominant mechanism toward abrasive wear. The deformational component is controlled by bulk mechanical properties (modulus, hardness, yield stress). Two surfaces in contact only touch at their asperities, so the true contact area is far smaller than the apparent geometric area. Because carbon fibers are significantly stiffer and harder than the polymer matrix, their addition increases the elastic modulus, yield stress and hardness of the material, which reduces the contact area and consequently both the frictional force and the susceptibility to surface damage. This mechanical argument underpins all CF-related tribological improvements reported in the literature [99].

#### 4.3.1. Friction Performance

The primary mechanism of COF reduction is the formation of a carbonaceous transfer film on the counterface: fibers exposed at the wear surface deposit a thin graphitic layer during sliding, reducing direct polymer-to-sliding object contact and suppressing the adhesive friction component. Film continuity, and thus the magnitude of COF reduction, depends on fiber content, fiber-matrix adhesion, contact temperature, and sliding orientation.

Carbon fiber addition consistently reduces COF under dry sliding, but the magnitude varies strongly with matrix type (Table 2). In compliant, high-COF matrices, the effect is large: Luo et al. [100] showed that the addition of short carbon fibers into Nylon can reduce the friction coefficient (from 0.70 to 0.40), and Al Abir et al. [101] observed a comparable reduction for PLA (from 0.70 to 0.45). The apparent discrepancy between Zawadzki et al. [102] (only 7% reduction for PLA + 15 wt.% CF) and Al Abir et al. [101] for the same matrix is process-related: Zawadzki et al. demonstrated that printing temperatures outside 190–220 °C degrade bead quality and prevent coherent film formation, confirming that tribological response in FFF composites is not purely a material property but is process-dependent. For engineering-grade matrices with intrinsically better tribological performance, the benefit is more modest: Dhakal et al. [103] reported only 11% decrease in COF for PEEK (0.35 to 0.31) where CF’s primary role is stabilizing, rather than substantially reducing, the transfer film. Two further results are important. Under water lubrication, both neat PEEK and PEEK + 10 wt.% CF converge to COF ≈ 0.12 [103]: the fluid assumes the lubricating role of the transfer film, eliminating the dry-sliding advantage of the fibers. This confirms that the friction benefit of carbon fibers is a self-lubricating capability, specific to dry or marginally lubricated conditions. Additionally, Lv et al. [104] reported orientation-dependent COF in PEEK-CF (0.24 normal vs. 0.28 parallel), arising because different sliding orientations expose different fiber-surface configurations and alter film continuity at bead boundaries, a process-induced anisotropy with no analog in injection-molded parts.

#### 4.3.2. Wear Behavior

Wear in 3D-printed CFRPs is a multi-step process involving matrix abrasion, fiber-matrix debonding, and eventual delamination of the printed layers, and it is strongly affected by the presence of carbon fibers, as well as weak points, such as defects, porosity, poor adhesion between layers and fibers [105]. The introduction of CF shifts the dominant wear mechanisms from large-scale plastic deformation toward localized fiber-matrix debonding and micro-delamination. Dhakal et al. [103] documented this phenomenon clearly for PEEK: the wear mechanism changed, but the dry-sliding wear rate did not (≈2 × 10^−6^ mm^3^/Nm for both neat and CF-reinforced). Interestingly, the authors showed that 3D printed neat/composite PEEK under dry sliding showed comparable specific wear rates to injection-molded neat PEEK.

Cui et al. [106] demonstrated that PEEK reinforced with short carbon fibers shows a significant reduction in COF (21–24%) and wear rate (from 4 to 0.12 × 10^−5^ mm^3^/Nm) under water-lubricated conditions, attributing it to improved load-bearing capacity and formation of a graphitized surface layer that suppresses adhesive wear.

Luo et al. [100] and Al Abir et al. [101] reported order-of-magnitude reductions in wear rate for Nylon and PLA, respectively (Figure 11). Nylon and PLA have poor intrinsic wear resistance, so carbon fiber hardness increase and load-bearing contribution produce large absolute gains; PEEK shows an intrinsically good wear resistance where these contributions can be marginal under specific conditions. A limitation specific to SCF composites is that frictional heating can soften the matrix, allowing fibers to be extracted and re-enter the contact as hard abrasive particles, potentially reversing the wear benefit [100]. Good matrix-fiber interfacial adhesion is essential, since short fibers are more susceptible to extraction than continuous fibers. This risk is negligible for continuous CF composites, where fibers cannot be extracted, and highlights the importance of adequate fiber-matrix interfacial bonding in SCF systems.

Process-induced features can dominate the wear response: in fact, porosity could be the primary “weak spot” in the wear resistance of FFF parts [105,107]. Previous studies on unfilled 3D printed polymers showed that tribological behavior is strictly dependent on processing defects. For instance, Wang et al. [108] showed that, for unfilled PLA, lower printing temperatures result in poorer interlayer adhesion and a higher number of printing defects, increasing susceptibility to material removal during wear. Higher printing temperatures mitigate this effect by reducing defect density and improving interlayer adhesion.

Similarly, Karthick et al. [109] proved that increasing the infill density from 60% to 100% in PP-CF composites reduces the specific wear rate by approximately 41%. A fully dense structure provides the necessary mechanical support to the fibers, preventing them from being easily uprooted (fiber pull-out) by the shear forces of the sliding counterface. The wear rate is also a function of the printing direction [106]: anisotropy due to the relative orientations of the printed layers with respect to the sliding direction also determines the wear mechanism. Other factors influencing wear behavior were investigated by Man et al. [105]: the authors showed that wear performance sliding normally with respect to the fiber orientation is leading to high stress concentration on fibers, leading to fiber/matrix debonding and fiber breakage, therefore worsening the tribological performance. Similar results were recently observed in other works [110]. Kozior et al. [111] highlighted that the surface roughness, commonly also known as “staircase effect”, in FFF parts influences the initial linear wear during the running-in period. Thinner layers generally reduce this effect, leading to a quicker transition to steady-state wear. Some of the results reported in this section are summarized in Table 3. Wang et al. [112] identified printing temperature and raster angle as the dominant factors influencing the tribological behavior of PEEK composites. However, general conclusions regarding the effects of these parameters have yet to be established.

To sum up, three conclusions emerge consistently from the available data. First, CF reduces COF under dry sliding in all studied systems via transfer film formation; the benefit diminishes under external lubrication. Second, wear rate improvement is strongly matrix-dependent: large for compliant matrices (PLA, Nylon), less significant for high-performance matrices (PEEK) under dry sliding. Third, FFF process parameters (e.g., infill density, printing orientation, layer thickness, printing speed and temperature, as mentioned above) can outweigh material composition as determinants of wear resistance, making process control a prerequisite for tribological optimization. It is important to note that despite the growing number of studies on the quantification of tribological properties, the overall understanding remains limited, especially when compared to traditional manufacturing methods (for instance, injection molded parts [103]). Most existing research focuses on the fabrication and characterization of friction and wear mechanisms, with relatively little effort devoted to direct comparisons with samples produced through conventional manufacturing processes.

## 5. Post-Processing Effects

The implementation of post-processing techniques, such as thermal annealing, has emerged as a promising approach for improving the mechanical performance of 3D-printed composites, with the ultimate goal of matching the properties of carbon fiber-reinforced composites produced through conventional manufacturing methods. Post-processing techniques can represent a pivotal strategy to mitigate the inherent defects of Fused Filament Fabrication in carbon fiber-reinforced polymer composites, such as high porosity and suboptimal interlaminar bonding [113,114]. Thermal annealing consists in heating the printed components above the glass transition temperature (T_g_), molecular mobility is enhanced, promoting polymer chain diffusion across bead interfaces and effectively narrowing inter-layer gaps. In short carbon fiber composites, thermal treatments improve the fiber-matrix interface by closing gaps induced by polymer shrinkage, which enhances load transfer and overall mechanical performance [114]. Research on continuous fiber composites has demonstrated that optimized thermal annealing cycles can reduce porosity by approximately 87%, leading to a significant increase in interlaminar shear strength [113]. Overall, mechanical [115,116,117] and tribological [118] properties could benefit from post-processing thermal treatment [105,106,107]. Hot isostatic pressing (HIP), a thermal treatment method utilizing both elevated temperature and isostatic pressure inside a vessel, has emerged as an additional post-processing technique to reduce porosity and voids thus increasing the interfacial area between layer-layer and matrix fiber. Some research [119,120,121,122,123,124] have demonstrated that HIP treatment is useful to improve mechanical performance, in terms of tensile modulus and strength due to the reduction of voids. However, it is worth noting that the temperature, pressure and time of the treatment must be properly tuned to achieve an improvement in the mechanical properties: Xu et al. [120] showed that long annealing times or high annealing temperatures lead to embrittlement due to a marked increase in crystallinity, despite a concurrent reduction in void content. Recently, chemical treatments (either liquid or vapor), utilizing specific chemicals for the material to be 3D-printed, were shown to improve the tensile strength, flexural strength, Shore D hardness, and surface roughness [116,117]. Ultrasonic treatments have also been applied to improve fiber-matrix interfacial bonding [125], addressing poor fiber-matrix adhesion (pull-out failure mode) discussed above. Despite the interesting preliminary results, there is still limited research regarding the role of post-processing techniques, especially chemical and ultrasonic treatments, on the overall mechanical and tribological performance of 3D printed CFRP composites. This is an area that will most likely be substantially explored in the coming years.

## 6. Future Trends

The following section identifies the key emerging research directions in FFF-printed CFRPs:

1. Hybrid reinforcing systems

A promising strategy to overcome the geometric and mechanical limitations inherent to FFF-printed CFRPs is the development of hybrid reinforcement systems, such as short/continuous CF combinations or carbon/glass hybrid fiber composites [126]. By strategically pairing CFs with complementary reinforcements, recent studies have sought to exploit synergistic effects that individual fiber systems cannot achieve alone.

For instance, by combining the performance advantages of continuous and short CFs, the continuous CFs provide the necessary structural stiffness and tensile strength, while short fibers help improve the bonding performance between adjacent filaments [127]. Notably, previous studies found that synergistic reinforcement of FFF-printed CFRPs with both short and continuous CFs was superior to composites with individual fiber reinforcement for tensile strength, though a similar improvement was not observed for the elastic modulus [128]. However, the mechanical properties of FFF-printed hybrid components remain significantly lower than those of the original printing filaments [129]. This performance degradation is mainly attributed to an increase in void content, a reduction in the overall density of the material, and poor fusion at the interlayer interfaces. These processing-induced flaws leave FFF-printed hybrid CFRPs inferior to conventionally manufactured counterparts, and post-treatments such as thermal annealing remain necessary to close the performance gap [130].

2. Closed-loop recycling and sustainable circularity: given the growing environmental and economic imperatives, establishing closed-loop recycling strategies for FFF-printed CFRPs represents a paramount trend [131,132,133,134]. Current research focuses on using recycled CFs into 3D-printed CFRPs while maintaining competitive mechanical properties. For example, using reclaimed CFs from pyrolysis of CFRP waste to reinforce PA6 matrix for FFF printing. However, to achieve performance comparable to products printed with virgin CFs, this requires the reclaimed CFs to have minimal residual char and no signs of surface damage [132]. An alternative approach involves directly remanufacturing FFF-printed continuous CFRPs by reprocessing printed components into polymer-impregnated CF filament, which are subsequently reused as printing feedstock to close the material loop without significant downgrading of fiber quality [131].
3. Artificial Intelligence (AI) implementation: Hybrid, physics-informed Machine Learning (ML) frameworks are being used for property prediction and process optimization in FFF-printed CFRPs. Nawafleh and AL-Oqla [135] showed that lightweight adaptive neuro-fuzzy inference system (ANFIS) architectures can reliably predict flexural behavior across experimentally accessible and inaccessible fiber fractions; Almeida and Gomes [136] coupled artificial neural network (ANN) surrogates for simultaneous multi-objective optimization of interlaminar strength and printing time; and Monticeli et al. [137] combined ANN with analysis of variance to map FFF process parameters to flexural response with sub-1% error. EisaZadeh et al. [138] demonstrated generative adversarial network (GAN)-assisted synthetic data generation as near-real-time FEA surrogates. Collectively, these works reflect a broader trend shift toward data-driven, experimentally validated frameworks capable of navigating the complex processing-structure-property relationships of FFF-printed composite systems.

## 7. Conclusions

This review examines the role of carbon fibers in FFF-printed composites, covering mechanical, fracture, and tribological performance. Tensile properties improve with CF addition, but the enhancement is strongly anisotropic and falls well below theoretical predictions. The gap between observed and predicted performance is not a fundamental limitation of the reinforcement mechanism but a consequence of fiber shortening during extrusion, non-ideal orientation distributions, and void formation, all of which are addressable through process optimization.

Fracture behavior presents a genuine duality: CF addition can either toughen or embrittle the matrix depending on fiber length relative to the critical fiber length and the quality of the fiber-matrix interface. Continuous fiber systems are competitive with conventionally manufactured composites under Mode I loading; short fiber systems carry a real embrittlement risk when interfacial adhesion is inadequate. This distinction has direct implications for material selection and process design in structural applications.

Tribologically, CF reduces friction under dry sliding through transfer film formation, with the magnitude of improvement governed by the matrix’s intrinsic tribological behavior. Wear rate improvements follow the same matrix-dependence. Crucially, process-induced defects, particularly porosity, can dominate the tribological response, reinforcing the conclusion that print quality is a prerequisite for realizing the material’s potential.

Post-processing, particularly thermal annealing, partially closes the performance gap with conventional manufacturing by reducing void content and improving interfacial consolidation, and represents a practical near-term strategy for improving part quality.

Significant gaps remain: the tribological behavior of continuous CF FFF composites is largely uncharacterized, scratch resistance has received almost no attention, and direct performance comparisons with injection-molded counterparts are scarce. These gaps, alongside the emerging directions of hybrid reinforcement, closed-loop recycling, and data-driven process optimization, define the frontier of this field.

## Figures and Tables

**Figure 1:**
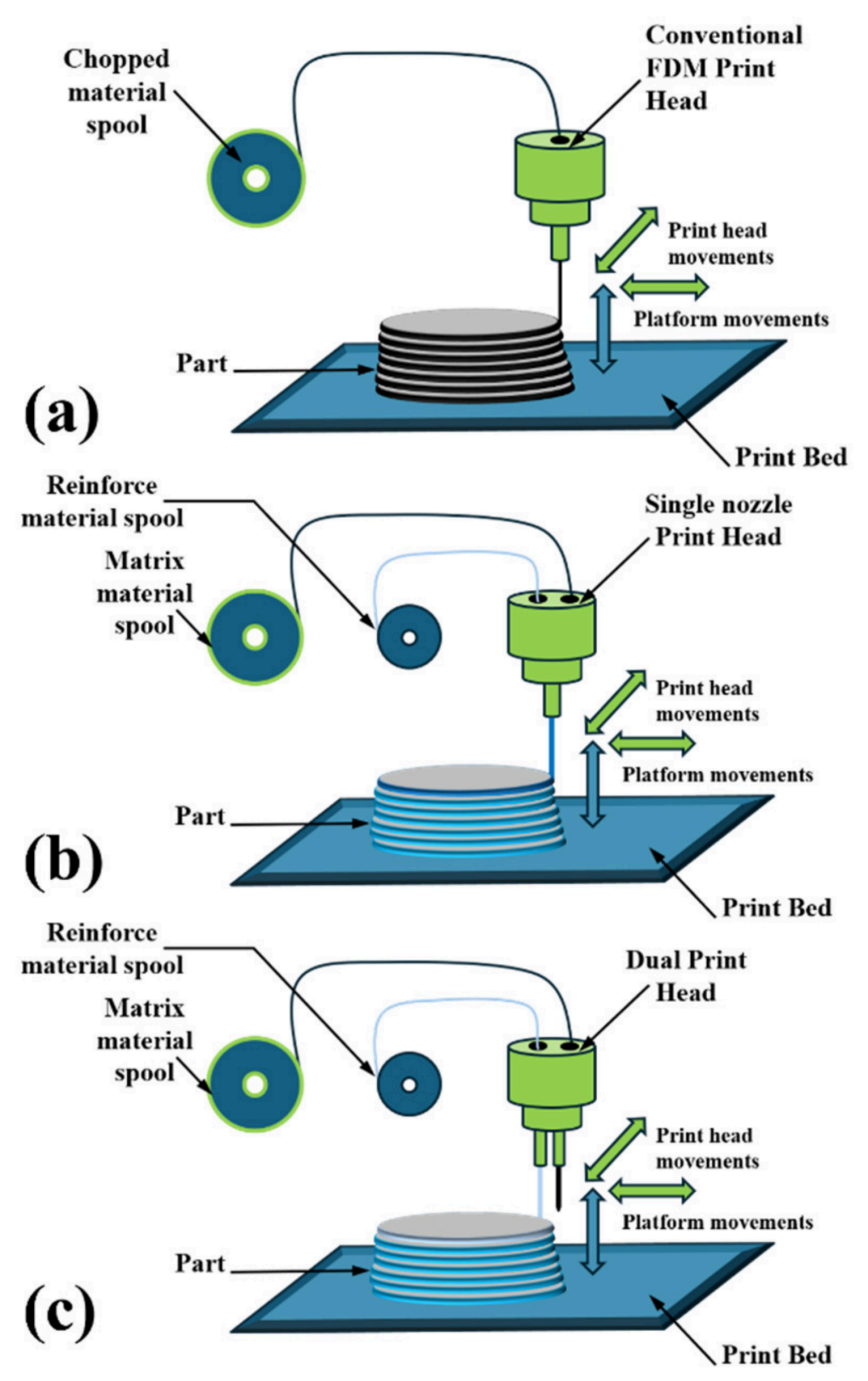
Schematic illustration of the fiber-reinforced FFF types (**a**) fiber is incorporated before the printing process (**b**) fiber is incorporated within the FFF print head (**c**) fiber is incorporated directly into the component. Figure created by the authors, conceptually informed by [24].

**Figure 2:**
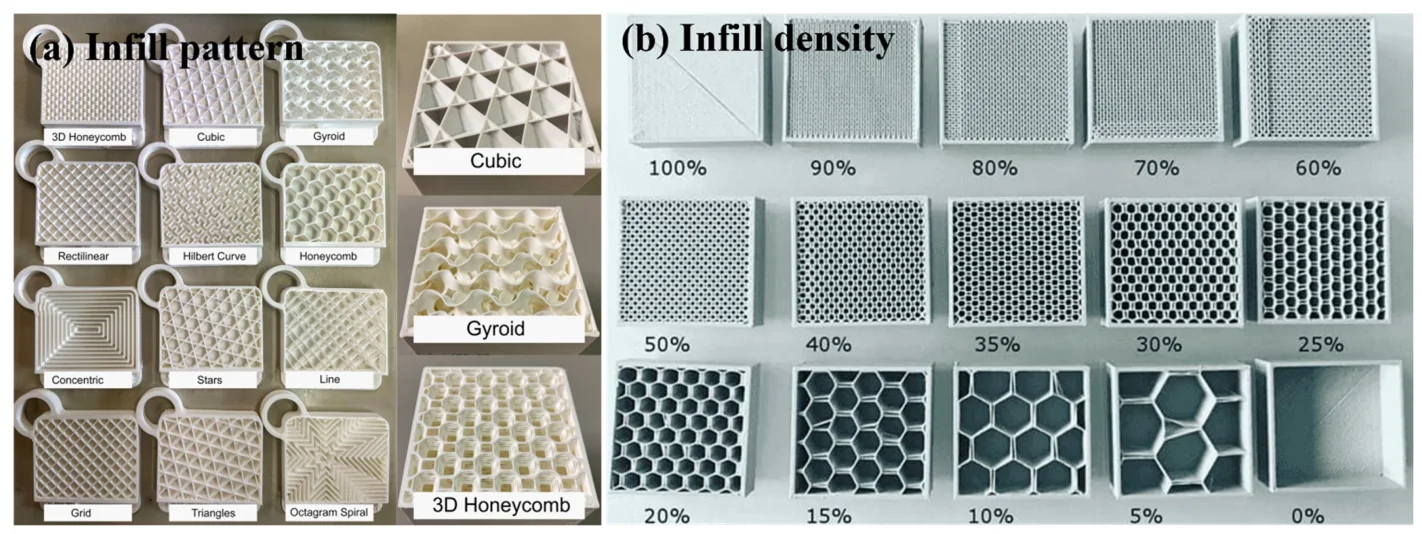
Examples of infill patterns and densities: (**a**) infill patterns [31], and (**b**) infill densities [37].

**Figure 3:**
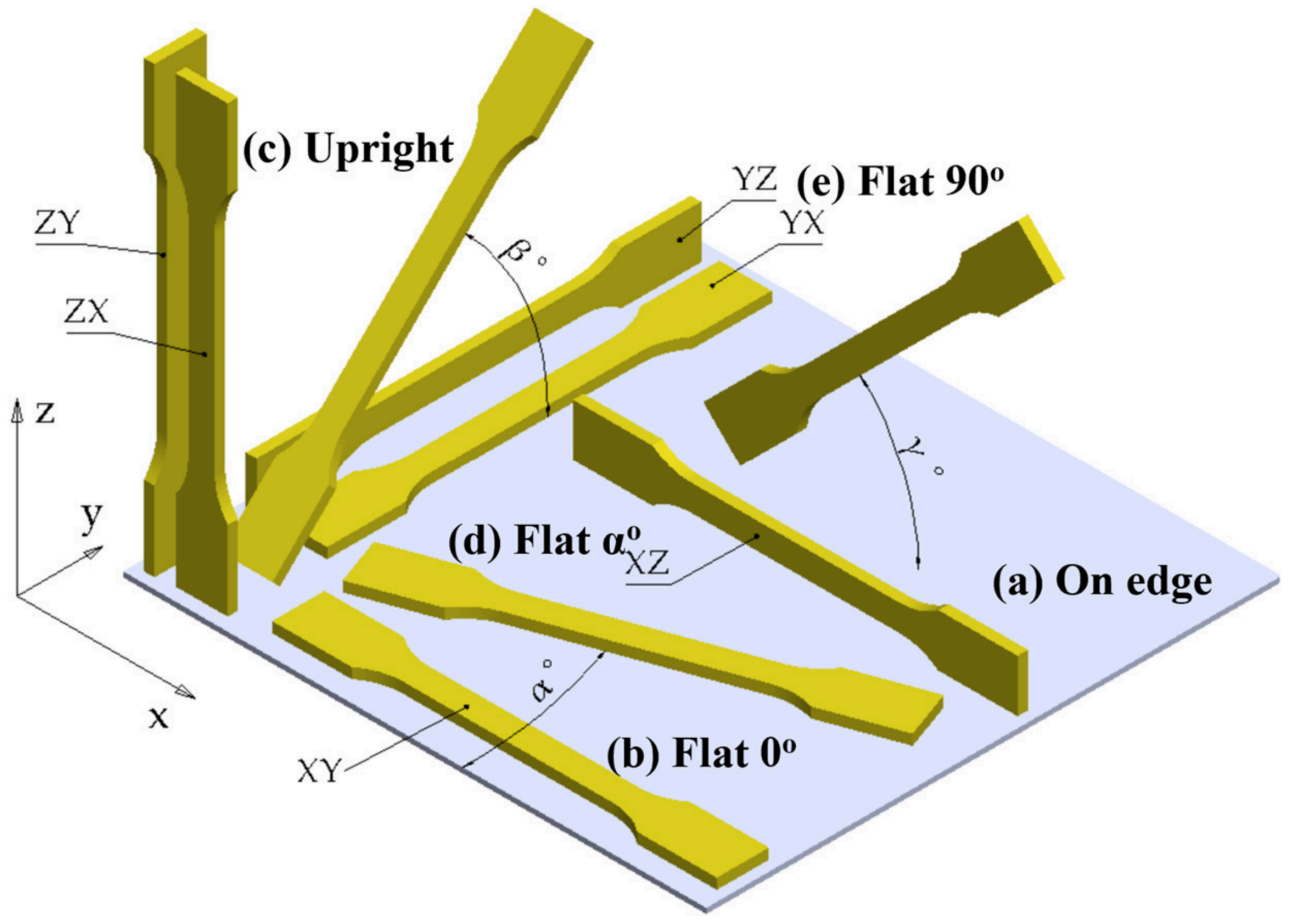
Tensile specimen oriented in various build orientations (**a**) On edge, (**b**) Flat 0°, (**c**) Upright, (**d**) Flat α^o^, (**e**) Flat 90° [40].

**Figure 4:**
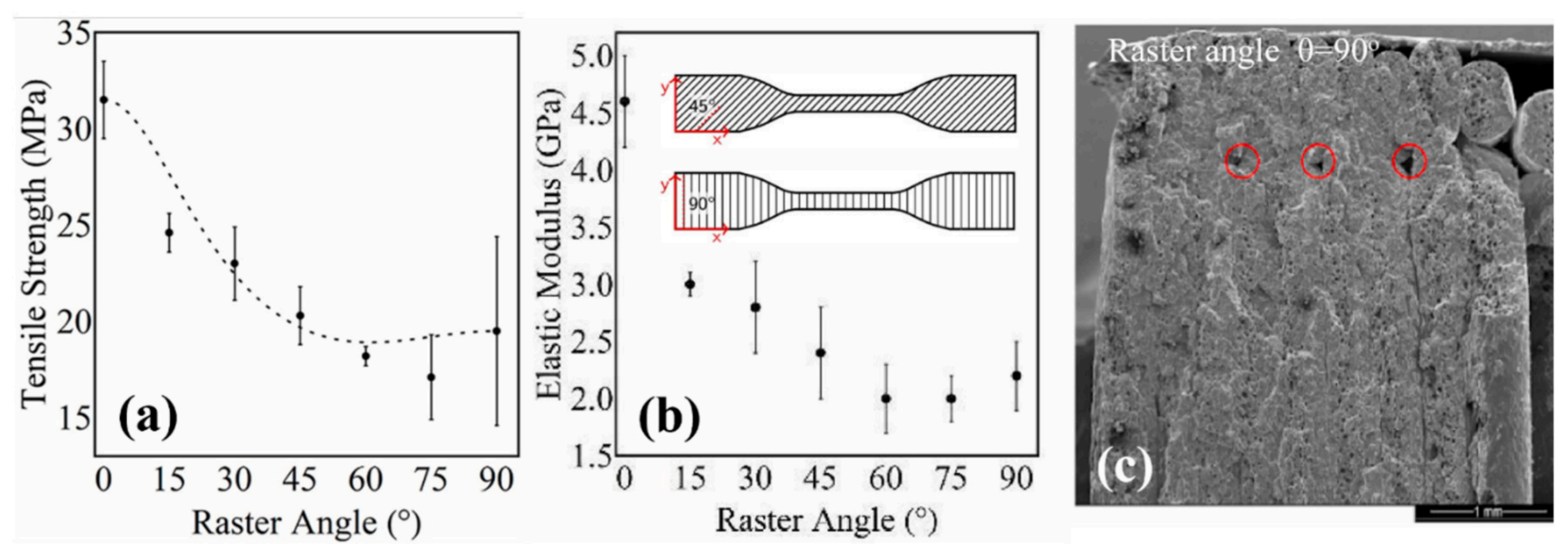
Variation of mechanical properties with raster angle (**a**) tensile strength, (**b**) elastic modulus of FFF-printed SCF-reinforced ABS. (**c**) SEM images of fracture surfaces of the tensile specimens at a raster angle of 90°. Red circles show some of the inter-bead porosity [44].

**Figure 5:**
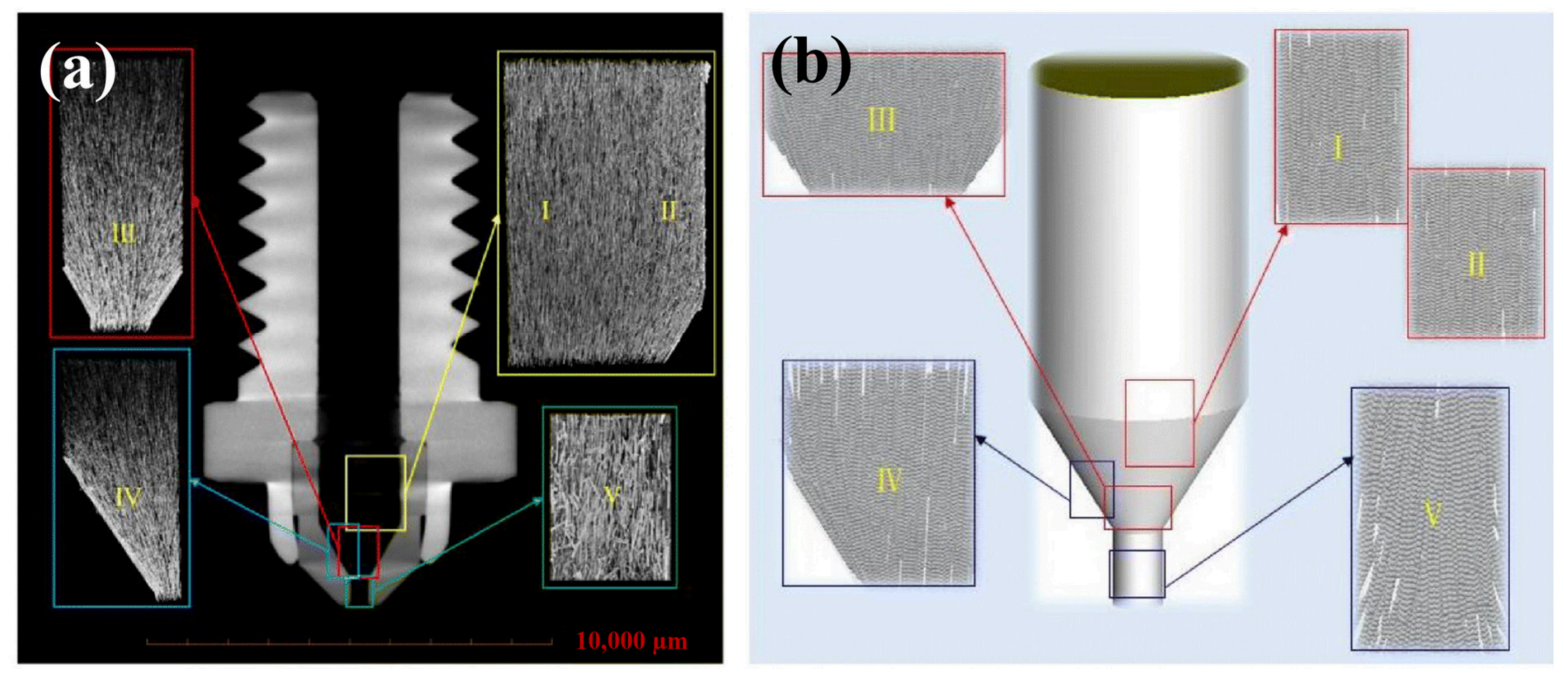
Short carbon fiber distributions in representative regions (I–V) of FFF-printed CF/PA6 composites: (**a**) X-ray microtomography images showing fiber distributions within and around the nozzle; (**b**) corresponding numerical simulation results [51].

**Figure 6:**
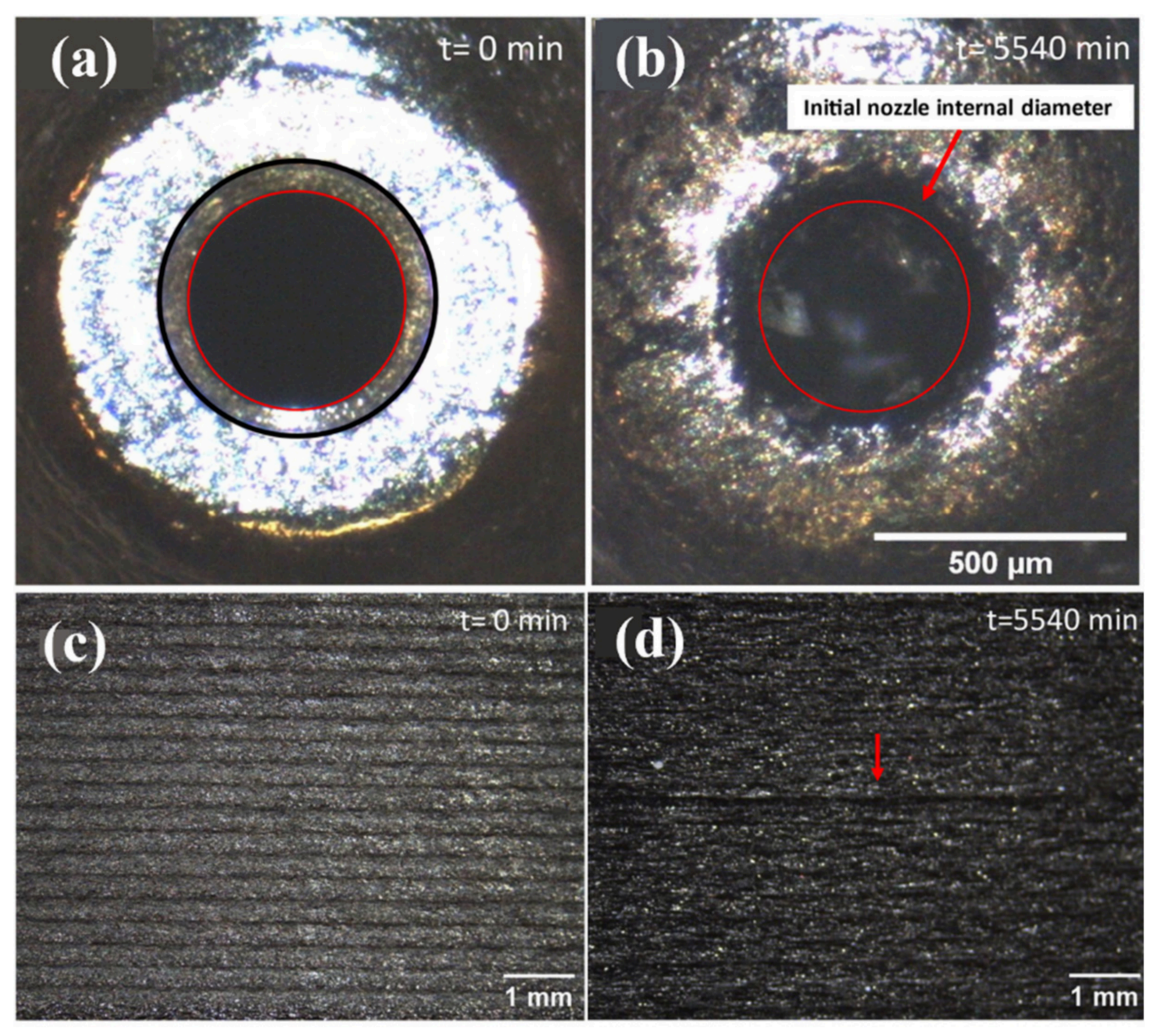
Optical microscopic images of the nozzle tip area at different printing time values: (**a**) t = 0 min, (**b**) t = 5540 min; Upper surfaces of FFF-printed Polyamide 6 matrix reinforced with 20 wt.% of SCFs (up to 250 µm in length) tensile specimens at different printing time values: (**c**) t = 0 min, (**d**) t = 5540 min [53]. In Figure 6b, the red circle marks the initial internal diameter of the nozzle, and the red arrow indicates the wear of the nozzle tip after 5540 minutes of printing. In Figure 6d, the red arrow indicates a macroscopic crack on the upper surface of the printed specimen.

**Figure 7:**
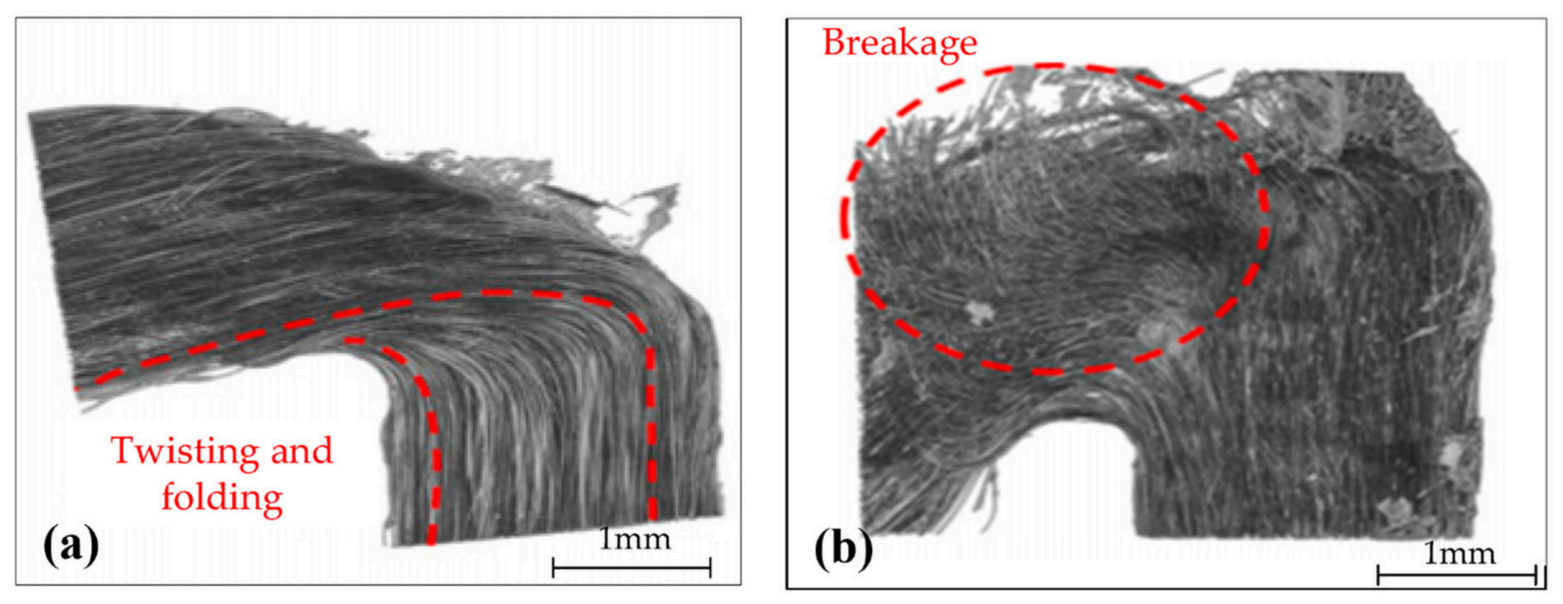
Morphological defects in 3D-printed continuous carbon fiber filaments at a bending region: (**a**) twisting and folding of fiber bundles; (**b**) fiber bundle breakage [56].

**Figure 8:**
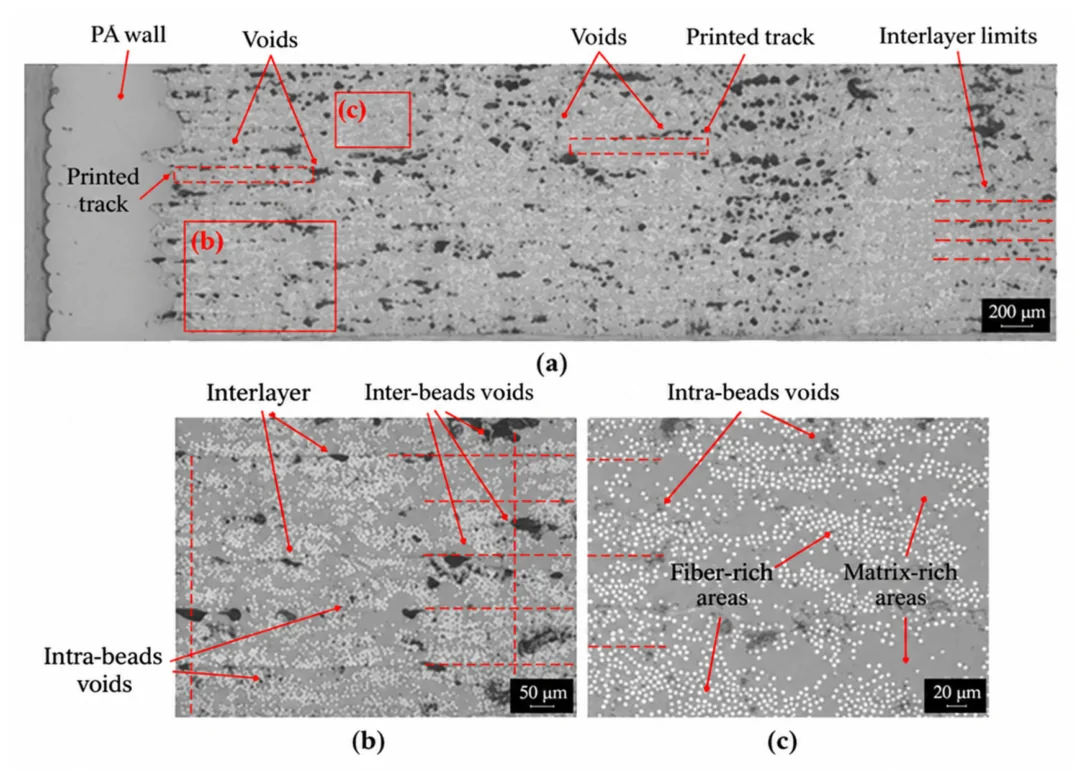
Optical images of polished cross-sections of (**a**) the CCF filament and (**b**) magnified view of a region with high fibers concentration within the CCF filament; (**c**) FESEM micrograph of the cryo-fractured surface of CCF tow [58].

**Figure 9:**
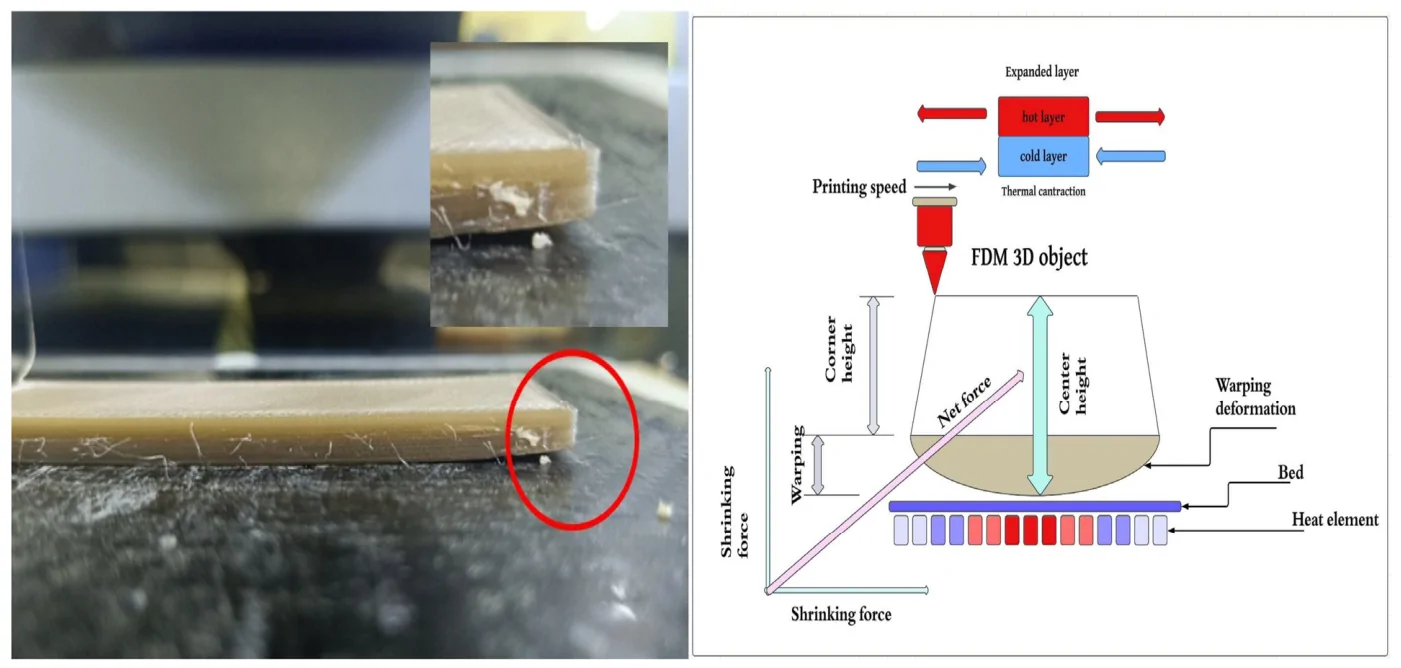
On the left: side view of the simulated warpage deformation of the PEEK component; on the right: schematic example of physics behind warpage phenomenon [62].

**Figure 10:**
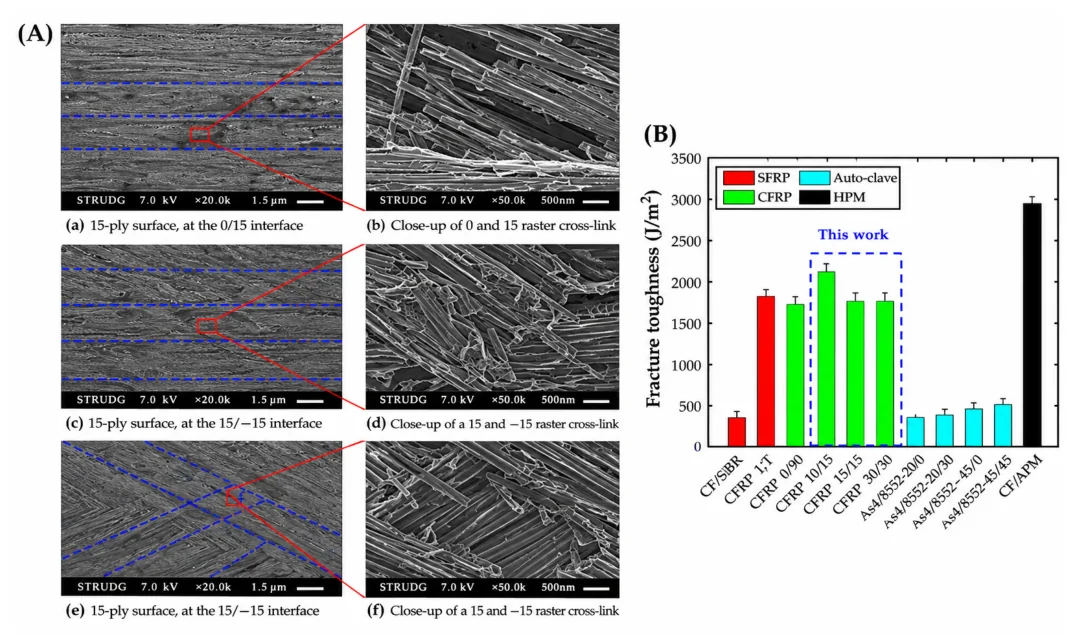
(**A**) Fractographic SEM images of the fracture surface, where blue lines indicate filament raster direction and (**B**) comparison of interlaminar fracture toughness of the results of the present work with (1) short fiber reinforced (SFRP, red columns) composites with 15wt.% [82], (2) unidirectional carbon fiber reinforced composites in Polyamide matrix (this work, [79]), (3) fiber reinforced polymer AS4/8552 (light blue columns) [84] and (4) Hot-Press Molded (HPM) using 3D printing materials [82]. Figures taken from [77].

**Figure 11:**
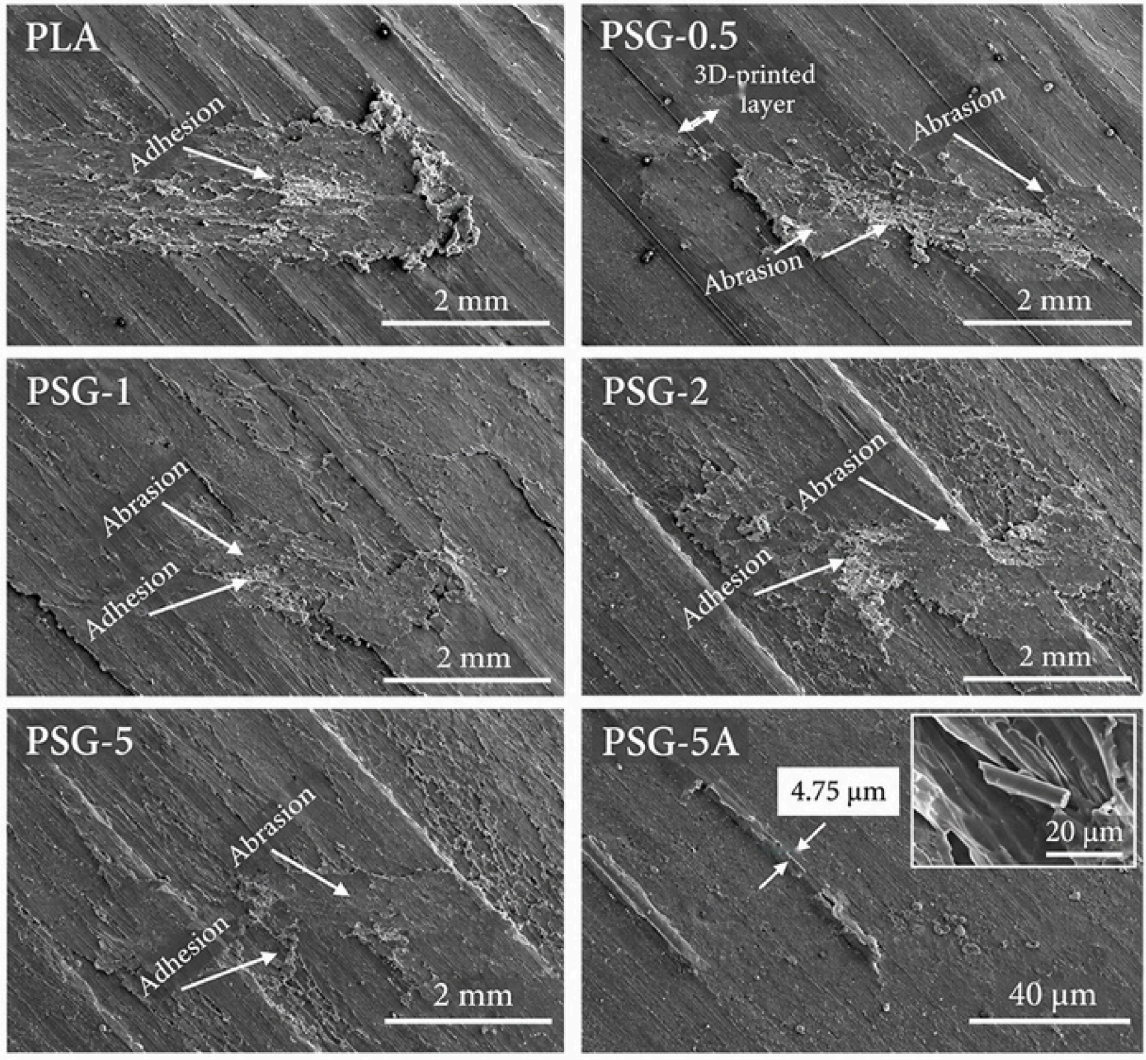
SEM images of the wear scars on the 3D-printed samples after the tribological tests. Tests were run by means of ball-on-disk setup, using a stainless steel (100Cr6) ball (5 mm diameter) and applying a normal load of 5 N with a stroke length of 6 mm and a frequency of 8.5 Hz [101].

**Table 1:** Representative quantitative effects of key FFF processing parameters on the mechanical properties of carbon fiber-reinforced polymer composites.

Processing Parameter	Material System	Parameter Range	Mechanical Response	Reference
Infill density	Short CF/ABS	20% → 40% → 60% → 80%	Tensile strength: 21.3 → 26.0 → 30.0 → 36.9 MPa	Turaka et al. [46]
Layer thickness	Continuous CF/PLA	0.25 → 0.3 → 0.35 → 0.4 mm	Flexural strength: 510 → 401 → 393 → 392 MPa	Zhang et al. [47]
Raster angle	Short CF/ABS	0° → 15°	Tensile strength: 31.5 → 24.6 MPa Young’s modulus: 4.6 → 3.0 GPa	Srinivasan Ganesh Iyer et al. [44]
Bed temperature	Short CF/PLA	60 °C → 70 °C → 80 °C	Tensile strength: 32.4 → 33.0 → 32.0 MPa	Lee et al. [48]
Printing temperature (liquefier/nozzle temperature)	Continuous CF/PLA	180 °C → 200 °C → 220 °C → 240 °C	Flexural strength: ≈116 → ≈140 → ≈150 → ≈155 MPa	Tian et al. [38]
Printing speed (nozzle traverse speed)	Continuous CF/PLA	400 → 600 → 800 → 1000 mm/min	Flexural strength: ≈132 → ≈102 → ≈90 → ≈85 MPa	Pappas et al. [49]

**Table 2:** Summary of the research work: this table summarizes the work, the material and carbon content, test condition and the variation in coefficient of friction (COF) for neat and reinforced systems.

Reference	Material	Conditions	COF [-]
Luo et al. [100]	Neat Nylon	Dry sliding	0.70
	Nylon + 9.6 wt.% CF		0.40
Zawadzki et al. [102]	Neat PLA	Dry sliding	0.46
	PLA + 15 wt.% CF		0.43
Al Abir et al. [101]	Neat PLA	Dry sliding	0.70
	PLA + 5vwt.% CF		0.45
Dhakal et al. [103]	Neat PEEK	Dry sliding	0.35
	PEEK + 10 wt.% CF		0.31
	Neat PEEK	Water lubricated	0.12
	PEEK + 10 wt.% CF		0.12

**Table 3:** Summary of the research work: this table summarizes the work, the material and carbon content, test conditions, and the variation in specific wear rate for neat and reinforced systems.

Reference	Material	Conditions	Specific Wear Rate [mm^3^/N·m]
Dhakal et al. [103]	Neat PEEK	Dry sliding	≈2 × 10^−6^
	PEEK + 10 wt.% CF		≈2 × 10^−6^
	Neat PEEK	Water lubricated	≈56 × 10^−6^
	PEEK + 10 wt.% CF		≈2 × 10^−6^
Cui et al. [106]	Neat PEEK	Water lubricated	≈4 × 10^−5^
	PEEK + 5 wt.% CF		≈0.12 × 10^−5^
Luo et al. [100]	Neat Nylon	Dry sliding	≈16 × 10^−6^
	Nylon + 9.6 wt.% CF		≈1 × 10^−6^

## Data Availability

No new data were created or analyzed in this study. Data sharing is not applicable to this article.

## References

[1] Hull C.W. (1986). Apparatus for Production of Three-Dimensional Objects by Stereolithography. U.S. Patent.

[2] De Noni L., Zorzetto L., Briatico-Vangosa F., Rink M., Ruffoni D., Andena L. (2022). Modelling the interphase of 3D printed photo-cured polymers. Compos. B Eng..

[3] Zorzetto L., Andena L., Briatico-Vangosa F., De Noni L., Thomassin J.M., Jérôme C., Grossman Q., Mertens A., Weinkamer R., Rink M. (2020). Properties and role of interfaces in multimaterial 3D printed composites. Sci. Rep..

[4] Ng W.L., Lee J.M., Zhou M., Chen Y.W., Lee K.X.A., Yeong W.Y., Shen Y.-F. (2020). Vat polymerization-based bioprinting—Process, materials, applications and regulatory challenges. Biofabrication.

[5] Chatham C.A., Long T.E., Williams C.B. (2019). A review of the process physics and material screening methods for polymer powder bed fusion additive manufacturing. Prog. Polym. Sci..

[6] Lupone F., Padovano E., Casamento F., Badini C. (2022). Process phenomena and material properties in selective laser sintering of polymers: A review. Materials.

[7] Cuan-Urquizo E., Barocio E., Tejada-Ortigoza V., Pipes R.B., Rodriguez C.A., Roman-Flores A. (2019). Characterization of the mechanical properties of FFF structures and materials: A review on the experimental, computational and theoretical approaches. Materials.

[8] Haryńska A., Carayon I., Kosmela P., Szeliski K., Łapiński M., Pokrywczyńska M., Kucińska-Lipka J., Janik H. (2020). A comprehensive evaluation of flexible FDM/FFF 3D printing filament as a potential material in medical application. Eur. Polym. J..

[9] Patterson A.E., Chadha C., Jasiuk I.M., Allison J.T. (2023). Tensile behavior of individual fibers and films made via material extrusion additive manufacturing. Next Mater..

[10] Townsend M.J., Lutonsky C., Vajipeyajula B., Asadi A., Tai B., Patterson A.E. (2026). Mechanical property degradation of additively manufactured polylactide produced from no-blend recycled feedstock. J. Thermoplast. Compos. Mater..

[11] Gonabadi H., Yadav A., Bull S.J. (2020). The effect of processing parameters on the mechanical characteristics of PLA produced by a 3D FFF printer. Int. J. Adv. Manuf. Technol..

[12] Ferreira R.T.L., Amatte I.C., Dutra T.A., Bürger D. (2017). Experimental characterization and micrography of 3D printed PLA and PLA reinforced with short carbon fibers. Compos. B Eng..

[13] Shi S., Deng S., Jiang Y., Chen J., Sporrer L., Cheng F., Guo Q., Jing J., Chen Y. (2026). Hierarchical Manufacturing of Anisotropic and High-Efficiency Electromagnetic Interference Shielding Modules for Smart Electronics. Nanomicro Lett..

[14] Vidakis N., Petousis M., Kechagias J.D. (2022). Parameter effects and process modelling of Polyamide 12 3D-printed parts strength and toughness. Mater. Manuf. Process..

[15] Vidakis N., Petousis M., Kechagias J.D. (2022). A comprehensive investigation of the 3D printing parameters’ effects on the mechanical response of polycarbonate in fused filament fabrication. Prog. Addit. Manuf..

[16] Szykiedans K., Credo W., Osiński D. (2017). Selected Mechanical Properties of PETG 3-D Prints. Procedia Eng..

[17] Dua R., Rashad Z., Spears J., Dunn G., Maxwell M. (2021). Applications of 3d-printed peek via fused filament fabrication: A systematic review. Polymers.

[18] Liu S., Shu T., Zhao Y., Zhao F., Song F., Hou X., Zhang Y., Liu C., Liu S., Sun C. (2026). Properties investigation on 3D-printed polyetherketoneketone (PEKK)—A proper candidate for bone replacement. Biomater. Adv..

[19] De Noni L., West S., Legendre H., Sue H.J., Patterson A.E. (2025). Mechanical Characterization of Additively Manufactured Poly(3-hydroxybutyrate-co-3-hydroxyhexanoate) Biopolymer. J. Appl. Polym. Sci..

[20] Rodzeń K., Harkin-Jones E., Wegrzyn M., Sharma P.K., Zhigunov A. (2021). Improvement of the layer-layer adhesion in FFF 3D printed PEEK/carbon fibre composites. Compos. Part A Appl. Sci. Manuf..

[21] Kantaros A., Soulis E., Petrescu F.I.T., Ganetsos T. (2023). Advanced Composite Materials Utilized in FDM/FFF 3D Printing Manufacturing Processes: The Case of Filled Filaments. Materials.

[22] Blok L.G., Longana M.L., Yu H., Woods B.K.S. (2018). An investigation into 3D printing of fibre reinforced thermoplastic composites. Addit. Manuf..

[23] Calignano F., Lorusso M., Roppolo I., Minetola P. (2020). Investigation of the mechanical properties of a carbon fibre-reinforced nylon filament for 3d printing. Machines.

[24] Naranjo-Lozada J., Ahuett-Garza H., Orta-Castañón P., Verbeeten W.M.H., Sáiz-González D. (2019). Tensile properties and failure behavior of chopped and continuous carbon fiber composites produced by additive manufacturing. Addit. Manuf..

[25] Pierson H.A., Celik E., Abbott A., De Jarnette H., Sierra Gutierrez L., Johnson K., Koerner H., Baur J.W. (2019). Mechanical Properties of Printed Epoxy-Carbon Fiber Composites. Exp. Mech..

[26] Chen A.Y., Baehr S., Turner A., Zhang Z., Gu G.X. (2021). Carbon-fiber reinforced polymer composites: A comparison of manufacturing methods on mechanical properties. Int. J. Lightweight Mater. Manuf..

[27] Batista M., Lagomazzini J.M., Ramirez-Peña M., Vazquez-Martinez J.M. (2023). Mechanical and Tribological Performance of Carbon Fiber-Reinforced PETG for FFF Applications. Appl. Sci..

[28] Stepashkin A.A., Chukov D.I., Senatov F.S., Salimon A.I., Korsunsky A.M., Kaloshkin S.D. (2018). 3D-printed PEEK-carbon fiber (CF) composites: Structure and thermal properties. Compos. Sci. Technol..

[29] Galos J., Hu Y., Ravindran A.R., Ladani R.B., Mouritz A.P. (2021). Electrical properties of 3D printed continuous carbon fibre composites made using the FDM process. Compos. Part A Appl. Sci. Manuf..

[30] Zhang Y., Zheng W., Wang Y., Ma K., Feng X., Ji Q., Guo W., Lu B. (2025). A review of 3D printing continuous carbon fiber reinforced thermoplastic polymers: Materials, processes, performance enhancement, and failure analysis. Polym. Compos..

[31] Lopes L., Reis D., Paula Junior A., Almeida M. (2023). Influence of 3D Microstructure Pattern and Infill Density on the Mechanical and Thermal Properties of PET-G Filaments. Polymers.

[32] Burnett C., Graninger G., Eren Z., Falzon B.G., Kazancı Z. (2025). Tensile performance of carbon fibre-reinforced 3D-printed polymers: Effect of printing parameters. Eng. Fail. Anal..

[33] Andrés González-Estrada O., Pertuz A., Quiroga J. (2018). Evaluation of Tensile Properties and Damage of Continuous Fibre Reinforced 3D-printed Parts. Key Eng. Mater..

[34] Qamar Tanveer M., Mishra G., Mishra S., Sharma R. (2022). Effect of infill pattern and infill density on mechanical behaviour of FDM 3D printed Parts—A current review. Mater. Today Proc..

[35] Ali Z., Yan Y., Mei H., Cheng L., Zhang L. (2023). Effect of infill density, build direction and heat treatment on the tensile mechanical properties of 3D-printed carbon-fiber nylon composites. Compos. Struct..

[36] El Essawi B., Abdallah S., Ali S., Nassir Abdo Mohammed A., Susantyoko R.A., Pervaiz S. (2024). Optimization of infill density, fiber angle, carbon fiber layer position in 3D printed continuous carbon-fiber reinforced nylon composite. Results Eng..

[37] ForgeLabs Advanced Manufacturing (2023). Infill Techniques for Making More Efficient FDM Parts. https://forgelabs.com/blog/infill-techniques-efficient-fdm-parts.

[38] Tian X., Liu T., Yang C., Wang Q., Li D. (2016). Interface and performance of 3D printed continuous carbon fiber reinforced PLA composites. Compos. Part A Appl. Sci. Manuf..

[39] Eryildiz M. (2021). Effect of Build Orientation on Mechanical Behaviour and Build Time of FDM 3D-Printed PLA Parts: An Experimental Investigation. Eur. Mech. Sci..

[40] Cojocaru V., Frunzaverde D., Miclosina C.O., Marginean G. (2022). The Influence of the Process Parameters on the Mechanical Properties of PLA Specimens Produced by Fused Filament Fabrication—A Review. Polymers.

[41] Ding Q., Li X., Zhang D., Zhao G., Sun Z. (2020). Anisotropy of poly(lactic acid)/carbon fiber composites prepared by fused deposition modeling. J. Appl. Polym. Sci..

[42] Yu S., Bale H., Park S., Hwang J.Y., Hong S.H. (2021). Anisotropic microstructure dependent mechanical behavior of 3D-printed basalt fiber-reinforced thermoplastic composites. Compos. B Eng..

[43] Pazhamannil R.V., Jishnu Namboodiri V.N., Govindan P., Edacherian A. (2022). Property enhancement approaches of fused filament fabrication technology: A review. Polym. Eng. Sci..

[44] Iyer S.G., Keles S. (2022). Effect of raster angle on mechanical properties of 3D printed short carbon fiber reinforced acrylonitrile butadiene styrene. Compos. Commun..

[45] Liu T., Tian X., Zhang M., Abliz D., Li D., Ziegmann G. (2018). Interfacial performance and fracture patterns of 3D printed continuous carbon fiber with sizing reinforced PA6 composites. Compos. Part A Appl. Sci. Manuf..

[46] Turaka S., Jagannati V., Pappula B., Makgato S. (2024). Impact of infill density on morphology and mechanical properties of 3D printed ABS/CF-ABS composites using design of experiments. Heliyon.

[47] Zhang Z., Long Y., Yang Z., Fu K., Li Y. (2022). An investigation into printing pressure of 3D printed continuous carbon fiber reinforced composites. Compos. Part A Appl. Sci. Manuf..

[48] Lee D., Wu G.Y. (2020). Parameters affecting the mechanical properties of three-dimensional (3D) printed carbon fiber-reinforced polylactide composites. Polymers.

[49] Pappas J.M., Thakur A.R., Leu M.C., Dong X. (2021). A parametric study and characterization of additively manufactured continuous carbon fiber reinforced composites for high-speed 3D printing. Int. J. Adv. Manuf. Technol..

[50] Croom B.P., Abbott A., Kemp J.W., Rueschhoff L., Smieska L., Woll A., Stoupin S., Koerner H. (2021). Mechanics of nozzle clogging during direct ink writing of fiber-reinforced composites. Addit. Manuf..

[51] Zhang H., Zhang L., Zhang H., Wu J., An X., Yang D. (2021). Fibre bridging and nozzle clogging in 3D printing of discontinuous carbon fibre-reinforced polymer composites: Coupled CFD-DEM modelling. Int. J. Adv. Manuf. Technol..

[52] Cai H., Chen Y. (2024). A Review of Print Heads for Fused Filament Fabrication of Continuous Carbon Fiber-Reinforced Composites. Micromachines.

[53] Bianchi I., Mancia T., Mignanelli C., Simoncini M. (2024). Effect of nozzle wear on mechanical properties of 3D printed carbon fiber-reinforced polymer parts by material extrusion. Int. J. Adv. Manuf. Technol..

[54] Heller B.P., Smith D.E., Jack D.A. (2016). Effects of extrudate swell and nozzle geometry on fiber orientation in Fused Filament Fabrication nozzle flow. Addit. Manuf..

[55] Zhang H., Chen J., Yang D. (2021). Fibre misalignment and breakage in 3D printing of continuous carbon fibre reinforced thermoplastic composites. Addit. Manuf..

[56] Liu M., Qu S., Li S., Yan X., Li W., Wang Y. (2025). Modeling and Compensation Methods for Trajectory Errors in Continuous Fiber-Reinforced Thermoplastic Composites Using 3D Printing. Polymers.

[57] Ning F., Cong W., Qiu J., Wei J., Wang S. (2015). Additive manufacturing of carbon fiber reinforced thermoplastic composites using fused deposition modeling. Compos. B Eng..

[58] Lupone F., Padovano E., Venezia C., Badini C. (2022). Experimental Characterization and Modeling of 3D Printed Continuous Carbon Fibers Composites with Different Fiber Orientation Produced by FFF Process. Polymers.

[59] Tekinalp H.L., Kunc V., Velez-Garcia G.M., Duty C.E., Love L.J., Naskar A.K., Blue C.A., Ozcan S. (2014). Highly oriented carbon fiber-polymer composites via additive manufacturing. Compos. Sci. Technol..

[60] Soete J., Badoux B., Swolfs Y., Gorbatikh L., Wevers M. Defect detection in 3D printed carbon fibre composites using X-ray Computed Tomography. Proceedings of the 9th Conference on Industrial Computed Tomography.

[61] Zhou J., Al-Akailah A., Ning F. (2025). Manufacturing Letters Prediction and Understanding of Warpage Generation during Additive Manufacturing of Continuous Carbon Fiber Composites. Manuf. Lett..

[62] Adarsh S.H., Nagamadhu M. (2025). Effect of Printing Parameters on Mechanical Properties and Warpage of 3D-Printed PEEK/CF-PEEK Composites Using Multi-Objective Optimization Technique. J. Compos. Sci..

[63] Melenka G.W., Cheung B.K.O., Schofield J.S., Dawson M.R., Carey J.P. (2016). Evaluation and prediction of the tensile properties of continuous fiber-reinforced 3D printed structures. Compos. Struct..

[64] Jiang D., Smith D.E. (2017). Anisotropic mechanical properties of oriented carbon fiber filled polymer composites produced with fused filament fabrication. Addit. Manuf..

[65] Yan X., Bethers B., Chen H., Xiao S., Lin S., Tran B., Jiang L., Yang Y. (2021). Recent Advancements in Biomimetic 3D Printing Materials With Enhanced Mechanical Properties. Front. Mater..

[66] Araya-Calvo M., López-Gómez I., Chamberlain-Simon N., León-Salazar J.L., Guillén-Girón T., Corrales-Cordero J.S., Sánchez-Brenes O. (2018). Evaluation of compressive and flexural properties of continuous fiber fabrication additive manufacturing technology. Addit. Manuf..

[67] Parmiggiani A., Prato M., Pizzorni M. (2021). Effect of the fiber orientation on the tensile and flexural behavior of continuous carbon fiber composites made via fused filament fabrication. Int. J. Adv. Manuf. Technol..

[68] Tóth C., Molnár K., Virág Á.D. (2025). Short fiber reinforcement in material extrusion 3D printing: A meta-analysis review with insights into sustainable alternatives. Polym. Compos..

[69] Saeed K., McIlhagger A., Harkin-Jones E., McGarrigle C., Dixon D., Ali Shar M., McMillan A., Archer E. (2022). Characterization of continuous carbon fibre reinforced 3D printed polymer composites with varying fibre volume fractions. Compos. Struct..

[70] Anwer A., Naguib H.E. (2018). Multi-functional flexible carbon fiber composites with controlled fiber alignment using additive manufacturing. Addit. Manuf..

[71] Fan C., Shan Z., Zou G., Zhan L., Yan D. (2021). Interfacial Bonding Mechanism and Mechanical Performance of Continuous Fiber Reinforced Composites in Additive Manufacturing. Chin. J. Mech. Eng. (Engl. Ed.).

[72] Liu P., Hou Z., Tian X., Zhu W., Wang C., He J., Li D. (2024). Evaluation and prediction of tensile properties of 3D-printed continuous carbon/Kevlar fiber-filled composites by coaxial hybrid process. Polym. Compos..

[73] Liu G.R. (1998). A step-by-step method of rule-of-mixture of fiber-and particle-reinforced composite materials. Compos. Struct..

[74] Chen H., Bajaj D., Patil D., Zhu Z., Verghese N., Sue H.J. (2024). Fracture behavior of high-performance carbon fiber composites toughened by reactive polyetherimide. Polym. Compos..

[75] Avanzini A., Battini D., Giorleo L. (2022). Finite element modelling of 3D printed continuous carbon fiber composites: Embedded elements technique and experimental validation. Compos. Struct..

[76] Liu B., Dong B., Li H., Lou R., Chen Y. (2024). 3D printing finite element analysis of continuous fiber reinforced composite materials considering printing pressure. Compos. B Eng..

[77] Santos J.D., Guerrero J.M., Blanco N., Fajardo J.I., Paltán C.A. (2023). Numerical and Experimental Analysis of the Mode I Interlaminar Fracture Toughness in Multidirectional 3D-Printed Thermoplastic Composites Reinforced with Continuous Carbon Fiber. Polymers.

[78] Kong X., Luo J., Luo Q., Li Q., Sun G. (2022). Experimental study on interface failure behavior of 3D printed continuous fiber reinforced composites. Addit. Manuf..

[79] Santos J.D., Fernández A., Ripoll L., Blanco N. (2022). Experimental Characterization and Analysis of the In-Plane Elastic Properties and Interlaminar Fracture Toughness of a 3D-Printed Continuous Carbon Fiber-Reinforced Composite. Polymers.

[80] Iragi M., Pascual-González C., Esnaola A., Lopes C.S., Aretxabaleta L. (2019). Ply and interlaminar behaviours of 3D printed continuous carbon fibre-reinforced thermoplastic laminates; effects of processing conditions and microstructure. Addit. Manuf..

[81] Ogaili A.A.F., Basem A., Kadhim M.S., Al-Sharify Z.T., Jaber A.A., Njim E.K., Al-Haddad L.A., Hamzah M.N., Al-Ameen E.S. (2024). The Effect of Chopped Carbon Fibers on the Mechanical Properties and Fracture Toughness of 3D-Printed PLA Parts: An Experimental and Simulation Study. J. Compos. Sci..

[82] Young D., Wetmore N., Czabaj M. (2018). Interlayer fracture toughness of additively manufactured unreinforced and carbon-fiber-reinforced acrylonitrile butadiene styrene. Addit. Manuf..

[83] Friedrich K. (1985). Microstructural Efficiency and Fracture Toughness of Short Fiber/Thermoplastic Matrix Composites. Compos. Sci. Technol..

[84] Sebaey T.A., Blanco N., Costa J., Lopes C.S. (2012). Characterization of crack propagation in mode I delamination of multidirectional CFRP laminates. Compos. Sci. Technol..

[85] Somireddy M., Singh C.V., Czekanski A. (2020). Mechanical behaviour of 3D printed composite parts with short carbon fiber reinforcements. Eng. Fail. Anal..

[86] Papon E.A., Haque A. (2018). Tensile properties, void contents, dispersion and fracture behaviour of 3D printed carbon nanofiber reinforced composites. J. Reinf. Plast. Compos..

[87] Papon E.A., Haque A. (2019). Fracture toughness of additively manufactured carbon fiber reinforced composites. Addit. Manuf..

[88] Gao J., Li B., Bu H., Li X., Zhan X. (2023). Effect of infill density and reinforced perimeters on tensile properties and fracture mechanism of 3D printing carbon fiber–reinforced composite. Int. J. Adv. Manuf. Technol..

[89] Yavas D., Zhang Z., Liu Q., Wu D. (2021). Fracture behavior of 3D printed carbon fiber-reinforced polymer composites. Compos. Sci. Technol..

[90] Boğa C. (2021). Investigation of mechanical and fracture behavior of pure and carbon fiber reinforced ABS samples processed by fused filament fabrication process. Rapid Prototyp. J..

[91] PAscoe M.W., Tabor D. (1956). The friction and deformation of polymers. Math. Phys. Sci..

[92] Cohen S.C., Tabor D. (1966). The friction and lubrication of polymers. Proc. R. Soc. Lond. A Math. Phys. Sci..

[93] Lancaster J.K. (1990). Material-specific wear mechanisms: Relevance to wear modelling. Wear.

[94] Lancaster J.K. (1969). Abrasive Wear of Polymers. Wear.

[95] Tabor D. (1970). The hardness of solids. Rev. Phys. Technol..

[96] De Noni L., Mullins M., Sue H.-J. (2026). Effect of temperature on scratch and mar behaviors and visibility of amorphous thermoplastics. Polymer.

[97] De Noni L., Zhu X., Andena L., Noh K., Li Y., Vollenberg P., Sue H. (2024). Effect of physical aging on scratch behavior of polycarbonate. J. Appl. Polym. Sci..

[98] De Noni L., Marjuban S.M.H., Andena L., Noh K., Li Y., Vollenberg P., Sue H. (2023). Effect of color on scratch and mar visibility of polymers. J. Appl. Polym. Sci..

[99] Tabor D. (1948). A simple theory of static and dynamic hardness. Proc. R. Soc. Lond. A Math. Phys. Sci..

[100] Luo M., He Q., Wang H., Chang L. (2021). Tribological behavior of surface textured short carbon fiber- reinforced nylon composites fabricated by three-dimensional printing techniques. J. Tribol..

[101] Al Abir A., Chakrabarti D., Trindade B. (2023). Fused Filament Fabricated Poly(lactic acid) Parts Reinforced with Short Carbon Fiber and Graphene Nanoparticles with Improved Tribological Properties. Polymers.

[102] Zawadzki P., Rybarczyk J., Patalas A., Wierzbicka N., Łabudzki R., Baila D., Fodchuk I., Bonilla M. (2026). Tribological Properties of Fused Deposition Modeling-Printed Polylactic Acid and PLA-CF: Extrusion Temperature and Internal Structure. J. Tribol..

[103] Dhakal N., Espejo C., Morina A., Emami N. (2024). Tribological performance of 3D printed neat and carbon fiber reinforced PEEK composites. Tribol. Int..

[104] Lv X., Yang S., Pei X., Zhang Y., Wang Q., Wang T. (2024). Tribological anisotropy of PEEK composites filled with highly oriented carbon fibers manufactured by fused deposition modeling. Polym. Compos..

[105] Man Z., Wang H., He Q., Kim D.E., Chang L. (2021). Friction and wear behaviour of additively manufactured continuous carbon fibre reinforced PA6 composites. Compos. B Eng..

[106] Cui Z., Aiyiti W., Kasimu A., Dong L., Jia R., Shuai C. (2025). Improving tribological performance of 3D-printed PEEK and CFR-PEEK composites by combining optimized deposition strategies and post-processing conditions. Wear.

[107] Dhakal N., Wang X., Espejo C., Morina A., Emami N. (2023). Impact of processing defects on microstructure, surface quality, and tribological performance in 3D printed polymers. J. Mater. Res. Technol..

[108] Wang C., He Y., Lin Z., Zhao X., Sun C., Guo R., Wang X., Zhou F. (2024). Mechanical and tribological properties of FDM-printed polyamide. Tribol. Int..

[109] Karthick N., Soundararajan R., Arul R., Prasanth J.A. (2024). Evolution of Tribological Performance of Polypropylene with Carbon Fibre Composites Fabricated Through FDM Technology by Varying Infill Density. J. Inst. Eng. Ser. D..

[110] Xu Y., Zhao S., Hua C., Zhao Y., Lin L. (2026). Tribological anisotropy of 3D-printed PEEK composites: Effects of fiber orientation and multi-scale particle reinforcement. Tribol. Int..

[111] Kozior T., Hanon M.M., Zmarzły P., Gogolewski D., Rudnik M., Szot W. (2024). Evaluation of the influence of technological parameters of selected 3D printing technologies on tribological properties. 3D Print. Addit. Manuf..

[112] Wang C., Guo R., He Y., Yao X., Li G., Sun C., Wang X. (2026). Effects of 3D printing parameters on the mechanical and tribological properties of FDM-printed PEEK. Tribol. Int..

[113] Seok W., Jeon E., Kim Y. (2023). Effects of Annealing for Strength Enhancement of FDM 3D-Printed ABS Reinforced with Recycled Carbon Fiber. Polymers.

[114] Pascual-González C., San Martín P., Lizarralde I., Fernández A., León A., Lopes C.S., Fernández-Blázquez J. (2021). Post-processing effects on microstructure, interlaminar and thermal properties of 3D printed continuous carbon fibre composites. Compos. B Eng..

[115] Safari F., Kami A., Abedini V. (2022). 3D printing of continuous fiber reinforced composites: A review of the processing, pre- and post-processing effects on mechanical properties. Polym. Polym. Compos..

[116] Guduru K.K., Srinivasu G. (2020). Effect of post treatment on tensile properties of carbon reinforced PLA composite by 3D printing. Mater. Today Proc..

[117] Ali M.Y., Rao G.K.M., Prasad B.A. (2025). Experimental investigation of influence of post-processing variables on mechanical strength and surface quality of 3D printed carbon fiber reinforced polylactic acid samples. Prog. Addit. Manuf..

[118] Lin Z., Guo R., Lyu Y., Liu D., Zhu J., Wu L., Wang X. (2025). Enhanced interlayer adhesion and regulated tribological behaviors of 3D printed poly(ether ether ketone) by annealing. Tribol. Int..

[119] van de Werken N., Koirala P., Ghorbani J., Doyle D., Tehrani M. (2021). Investigating the hot isostatic pressing of an additively manufactured continuous carbon fiber reinforced PEEK composite. Addit. Manuf..

[120] Xu Z., Zou B., Ding S., Zhuang Y., Wang X. (2023). Study on the hot isostatic pressing post-treatment of FDM-3D printed continuous carbon fiber reinforced composites. J. Manuf. Process.

[121] Park S.J., Kim D.H., Ju H.G., Park S.J., Hong S., Son Y., Ahn I.H. (2023). Increased interlayer bonding strength of short carbon fiber composite fabricated by material extrusion via warm isostatic pressing (WIP) process. J. Mater. Res. Technol..

[122] Mei H., Ali Z., Yan Y., Ali I., Cheng L. (2019). Influence of mixed isotropic fiber angles and hot press on the mechanical properties of 3D printed composites. Addit. Manuf..

[123] Wang K., Tan Q., Wang J., Liu Y., Zhai Z., Yao S., Peng Y. (2024). A low-cost granular-medium hot quasi-isostatic pressing method for enhancing compressive properties of 3D printed structures. J. Manuf. Process.

[124] Jeon S., Park S.J., Moon S.K., Yang D. (2025). Enhanced mechanical properties and reduced anisotropy of material extrusion-manufactured short carbon fibre-reinforced plastic via cold isostatic pressing. Virtual Phys. Prototyp..

[125] Qiao J., Li Y., Li L. (2019). Ultrasound-assisted 3D printing of continuous fiber-reinforced thermoplastic (FRTP) composites. Addit. Manuf..

[126] Chen Y., Wei X., Mao J., Zhao M., Liu G. (2024). Experimental analysis of 3D printed continuous carbon/glass hybrid fiber reinforced PLA composites: Revealing synergistic mechanical properties and failure mechanisms. Polym. Compos..

[127] Xin Z., Ma Y., Chen Y., Wang B., Xiao H., Duan Y. (2023). Fusion-bonding performance of short and continuous carbon fiber synergistic reinforced composites using fused filament fabrication. Compos. B Eng..

[128] Peng Y., Wu Y., Wang K., Gao G., Ahzi S. (2019). Synergistic reinforcement of polyamide-based composites by combination of short and continuous carbon fibers via fused filament fabrication. Compos. Struct..

[129] Kong X., Sun G., Luo Q., Brykin V., Qian J. (2024). Experimental and theoretical studies on 3D printed short and continuous carbon fiber hybrid reinforced composites. Thin-Walled Struct..

[130] Handwerker M., Wellnitz J., Marzbani H., Tetzlaff U. (2021). Annealing of chopped and continuous fibre reinforced polyamide 6 produced by fused filament fabrication. Compos. B Eng..

[131] Tian X., Liu T., Wang Q., Dilmurat A., Li D., Ziegmann G. (2017). Recycling and remanufacturing of 3D printed continuous carbon fiber reinforced PLA composites. J. Clean. Prod..

[132] Su N., Pierce R.S., Rudd C., Liu X. (2022). Comprehensive investigation of reclaimed carbon fibre reinforced polyamide (rCF/PA) filaments and FDM printed composites. Compos. B Eng..

[133] Peng X., Zhang M., Guo Z., Sang L., Hou W. (2020). Investigation of processing parameters on tensile performance for FDM-printed carbon fiber reinforced polyamide 6 composites. Compos. Commun..

[134] Ateeq M., Shafique M., Azam A., Rafiq M. (2023). A review of 3D printing of the recycled carbon fiber reinforced polymer composites: Processing, potential, and perspectives. J. Mater. Res. Technol..

[135] Nawafleh N., AL-Oqla F.M. (2022). An innovative fuzzy-inference system for predicting the mechanical behavior of 3D printing thermoset carbon fiber composite materials. Int. J. Adv. Manuf. Technol..

[136] Almeida J.H.S., Gomes G.F. (2025). Optimising 3D-printed carbon fibre composites using machine learning: Balancing strength and efficiency. Mater. Des..

[137] Monticeli F.M., Neves R.M., Ornaghi H.L., Almeida J.H.S. (2022). Prediction of Bending Properties for 3D-Printed Carbon Fibre/Epoxy Composites with Several Processing Parameters Using ANN and Statistical Methods. Polymers.

[138] EisaZadeh H., Chatroudi N.H., Alizadeh R. (2026). AI-based prediction and numerical analysis of mechanical and microstructural parameters of carbon fiber reinforced 3D printed composites. Mater. Today Commun..

